# High-Mobility Flexible Transistors with Low-Temperature Solution-Processed Tungsten Dichalcogenides

**DOI:** 10.1021/acsnano.2c11319

**Published:** 2023-01-31

**Authors:** Tian Carey, Oran Cassidy, Kevin Synnatschke, Eoin Caffrey, James Garcia, Shixin Liu, Harneet Kaur, Adam G. Kelly, Jose Munuera, Cian Gabbett, Domhnall O’Suilleabhain, Jonathan N. Coleman

**Affiliations:** School of Physics, CRANN & AMBER Research Centres, Trinity College Dublin, Dublin D02 E8C0, Ireland

**Keywords:** electrochemical exfoliation, tungsten dichalcogenides, solution processing, transistors, Langmuir−Schaefer deposition

## Abstract

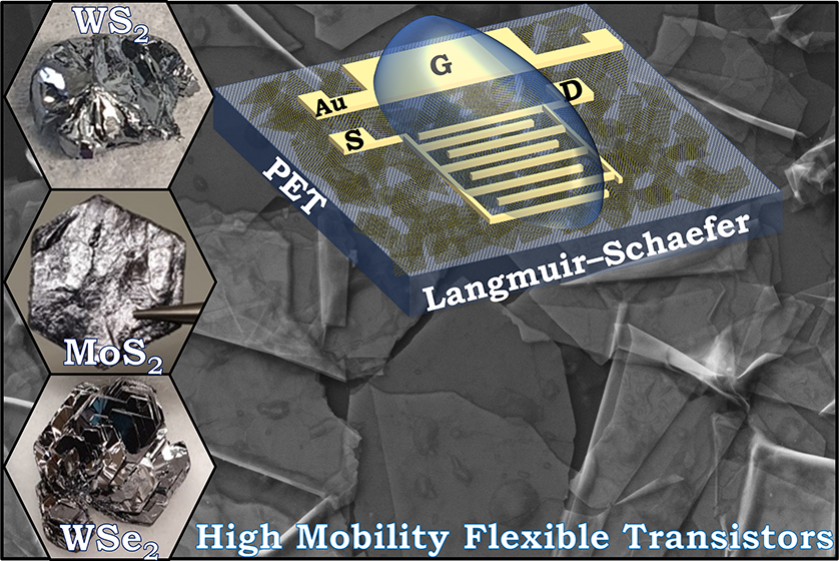

The investigation of high-mobility two-dimensional (2D) flakes beyond molybdenum disulfide (MoS_2_) will be necessary to create a library of high-mobility solution-processed networks that conform to substrates and remain functional over thousands of bending cycles. Here we report electrochemical exfoliation of large-aspect-ratio (>100) semiconducting flakes of tungsten diselenide (WSe_2_) and tungsten disulfide (WS_2_) as well as MoS_2_ as a comparison. We use Langmuir–Schaefer coating to achieve highly aligned and conformal flake networks, with minimal mesoporosity (∼2–5%), at low processing temperatures (120 °C) and without acid treatments. This allows us to fabricate electrochemical transistors in ambient air, achieving average mobilities of μ_MoS_2__ ≈ 11 cm^2^ V^–1^ s^–1^, μ_WS_2__ ≈ 9 cm^2^ V^–1^ s^–1^, and μ_WSe_2__ ≈ 2 cm^2^ V^–1^ s^–1^ with a current on/off ratios of *I*_on_/*I*_off_ ≈ 2.6 × 10^3^, 3.4 × 10^3^, and 4.2 × 10^4^ for MoS_2_, WS_2_, and WSe_2_, respectively. Moreover, our transistors display threshold voltages near ∼0.4 V with subthreshold slopes as low as 182 mV/dec, which are essential factors in maintaining power efficiency and represent a 1 order of magnitude improvement in the state of the art. Furthermore, the performance of our WSe_2_ transistors is maintained on polyethylene terephthalate (PET) even after 1000 bending cycles at 1% strain.

While electronic devices have traditionally been rigid, there is now a need for electronic components to conform to flexible substrates, while maintaining manufacturability in scale, to address new application areas in the automotive, healthcare, consumer electronics, and wearable electronics sectors.^1^ For example, flexible transistors are required for use in active matrix displays, sensors, and integrated circuits in many of these sectors.^2,3^ Solution processing of transistors has emerged as a method to manufacture flexible transistors using semiconducting inks, offering a broad material selection and considerable versatility, alongside low cost and reduced energy consumption over growth-based techniques. Over the past few decades, semiconducting inks of carbon nanotubes, organic polymers, and metal oxides have been studied. However, all have struggled to achieve transistor mobilities (μ) much beyond 10 cm^2^ V^–1^ s^–1^ at room temperature and in the ambient atmosphere required for digital electronics.^2,4^

2D flakes such as transition-metal dichalcogenides (TMDs) offer a route to exceed state-of-the-art transistor performances due to their high intrinsic mobility, μ > 50 cm^2^ V^–1^ s^–1^, reasonable stability under ambient conditions, and conformability to flexible substrates.^5,6^ Semiconducting inks of 2D flakes can be mass-manufactured by liquid-phase exfoliation (LPE) using techniques such as shear mixing or ultrasonication and deposited into networks of 2D flakes.^7^ However, achieving high-μ networks from LPE is challenging due to their relatively large flake thickness (up to 20 nm) and small lateral size (tens to hundreds of nanometers),^8^ which results in an unoptimized morphology (i.e., poor packing and alignment) on deposition.^9^ Furthermore, despite high mobility in the basal plane of the LPE 2D flakes,^10^ the network mobility has typically been limited by the interflake junctions,^9^ which can result in large hopping activation energies, *E*_a_ > 300 meV,^11^ leading to low μ in the range 0.01–0.3 cm^2^ V^–1^ s^–1^.^10,12−14^

Electrochemical exfoliation (EE) with quaternary ammonium molecules has emerged as an alternative route toward large 2D flakes, *L* > 1 μm, with a low *t* < 10 nm, which permits conformal junctions,^9^ reducing junction resistance (i.e., the electrical resistance at the interface between flakes) and *E*_a_ (<100 meV).^3,11^. To maximize network *μ* the flakes need to be conformal to each other and aligned (i.e. the network meso-porsity should be minimized < 5%).^9^ Deposition techniques which can help to align flakes, such as spin coating^15^ or Langmuir–Blodgett,^16^ have been used with EE MoS_2_ flakes on rigid Si/SiO_2_ to increase the transistor μ value to 1–8 cm^2^ V^–1^ s^–1^. However, in many cases, strategies such as acid treatment^15,17,18^ (e.g., bis(trifluoromethane)sulfonimide) or high-temperature annealing (>200 °C)^3,11,18,19^ are required to remove flake stabilization agents such as poly(vinylpyrrolidone) (PVP) or remove unintentional doping.^20^ Unfortunately, these strategies are typically incompatible with most flexible substrates, which require processing temperatures <120 °C to avoid deformation.^2^ The measurement of devices under vacuum (<10^–6^ mbar)^10,13,17,21^ or with passivation layers (e.g., aluminum oxide)^22^ has also been used to help reduce charge carrier scattering and thus ensure device μ > 0.1 cm^2^ V^–1^ s^–1^. However, vacuum processes can hinder applicability in a commercial environment, while passivation layers add additional complexity to the manufacturing process. Therefore, a protocol to yield high-mobility devices (μ > 1 cm^2^ V^–1^ s^–1^) that is compatible with flexible substrates and operational in ambient air without vacuum measurement, passivation layers, or acid treatments would be highly desirable. Furthermore, high-μ networks with TMDs have so far only been achievable with networks of MoS_2_ flakes despite the abundance of TMDs available to explore. Moreover, high μ > 1 cm^2^ V^–1^ s^–1^ networks of 2D flakes that go beyond MoS_2_ on a flexible substrate and in ambient air have not yet been achieved.

This work will utilize Langmuir–Schaefer deposition^23^ to explore flake networks of EE, WS_2_ and WSe_2_ (and MoS_2_ for comparison purposes), with minimal ink usage (<20 μL). Due to the high flake alignment and high aspect ratio (>100) the flakes have conformal junctions with minimal interflake junction resistance and therefore high μ on both Si/SiO_2_ and flexible PET in ambient air and without acid treatments or high-temperature annealing (>120 °C).

## Results and Discussion

### TMD Ink Production and Characterization

**Figure 1:**
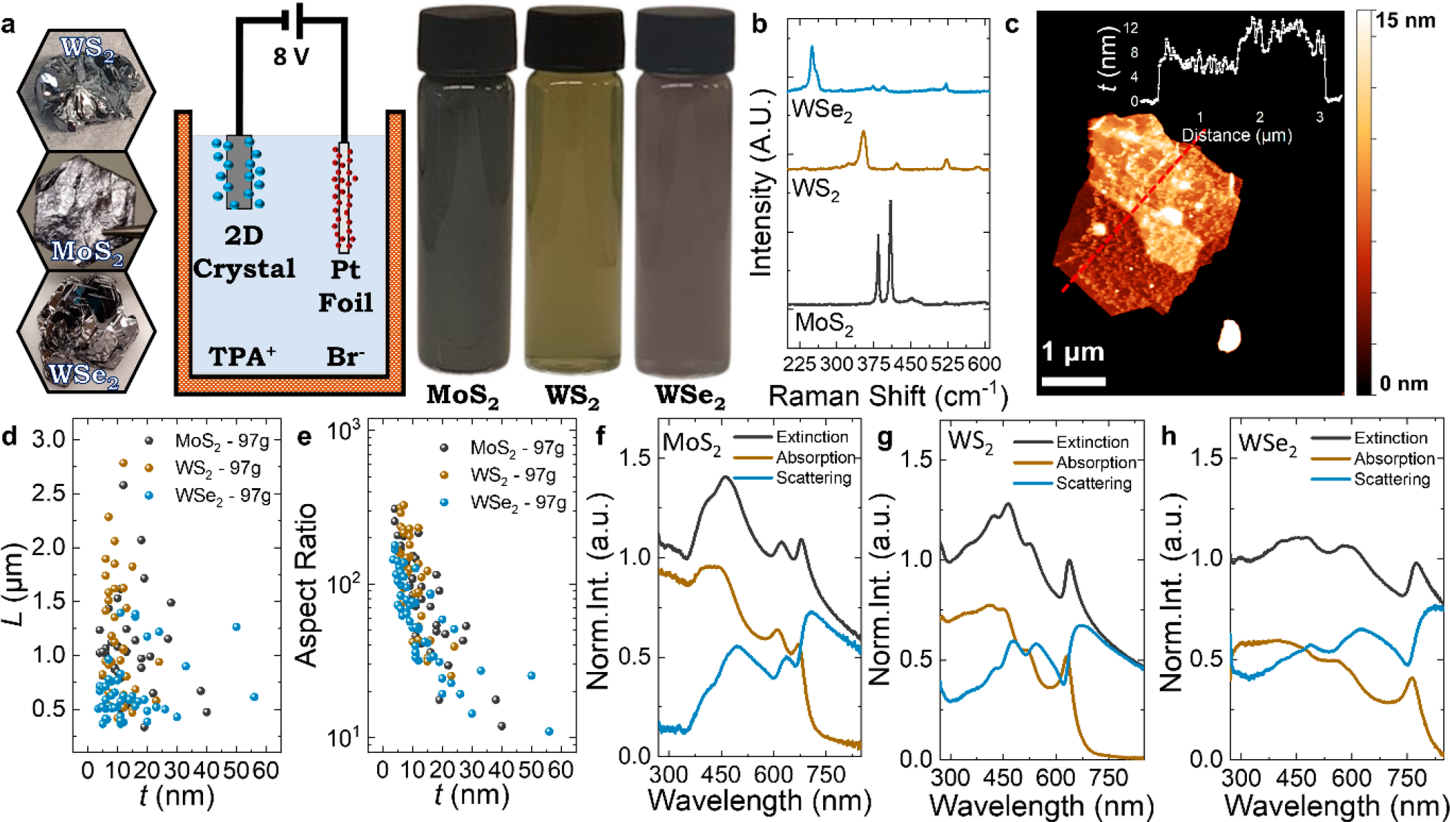
Electrochemical exfoliation of TMDs and their characterization. (a) Pictures of the bulk TMD crystals used for the electrochemical exfoliation, schematic of the intercalation with TPA^+^, cations and the resulting semiconducting inks after centrifugation. (b) Chemical analysis by Raman spectroscopy of the TMD flakes after exfoliation. (c) Atomic force microscopy micrograph of a MoS_2_ flake after centrifugation (97*g*). (d, e) Atomic force microscopy statistics of the flake AR and *L* and a function of the flake *t*. (f–h) Normalized optical characterization of the TMD inks by UV–vis showing the extinction, absorption, and scattering components as a function of wavelength shone through the ink.

We use EE to intercalate and expand 2D bulk crystals of MoS_2_, WS_2_ ,and WSe_2_ as shown in Figure 1a. Next, the intercalated TMD crystal is ultrasonically treated with PVP in DMF, centrifuged at 97*g*, and then solvent-exchanged into IPA after washing (see Methods) to form our MoS_2_, WS_2_, and WSe_2_ semiconducting inks (Figure 1a). The centrifugation washing is a key step to remove the residual PVP without annealing and ensure that the inks will be compatible with low processing temperatures of <120 °C. Raman spectroscopy is utilized to monitor TMD flake quality after exfoliation. Figure 1b depicts the spectra of the MoS_2_ (black), WS_2_ (brown), and WSe_2_ (blue) flakes and are consistent with previous reports of 2H semiconducting flakes since the J_2_ and J_3_ vibrational modes attributed to the metallic 1T phase are not observed (Supplementary Note 1 and Supplementary Figure 1a mark the absent peaks).^24−26^

Atomic force microscopy (AFM) statistics are used to estimate the lateral flake size (*L*) and apparent flake thickness (*t*) of the MoS_2_, WS_2_ and WSe_2_ flakes on Si/SiO_2_ substrates. Figure 1c is an AFM micrograph of a MoS_2_ flake with an associated cross section, while Figure 1d plots *L* versus *t* for individual flakes with no apparent correlation, which is unlike the case for LPE flakes (Supplementary Figure 1b).^8^ The average flake lengths, ⟨*L*⟩, were 1.0 ± 0.1, 1.2 ± 0.1, and 0.67 ± 0.05 μm, while the average apparent flake thicknesses, ⟨*t*⟩, were 14 ± 1, 10.5 ± 0.7, and 14 ± 1 nm for MoS_2_, WS_2_, and WSe_2_ flakes, respectively. Plotting the flake aspect ratio (AR, *L/t*) versus *t* in Figure 1e shows maximum AR values of 309, 326, and 177 for MoS_2_, WS_2_, and WSe_2_ flakes, respectively, with means of 102, 132, and 70. These values are significantly higher than ARs achieved with LPE by ultrasonication (AR ≈ 10–40),^8,27^ shear mixing (AR ≈ 4–40, Supplementary Note 1), cyclic shear mixing (AR ≈ 60)^28^ or microfluidization (AR ≈ 50).^29^ AR > 40 is required to make conformal flake-to-flake junctions to minimize the junction resistance to improve the device performance.^9^ It is known that the ratio of the in-plane-tearing energy to the out-of-plane-peeling energy determines the aspect ratio of liquid exfoliated flakes, as the former parameter controls the lateral size while the latter controls the nanosheet thickness.^8^ In electrochemical exfoliation, ion insertion is thought to reduce the peeling energy, thus increasing the aspect ratio.

In Figure 1f–h UV–visible optical absorption spectra of the MoS_2_, WS_2_, and WSe_2_ inks are taken with an integrating sphere to isolate the extinction, absorption, and scattering components of the TMD inks.^30^ The normalized spectra of the MoS_2_, WS_2_, and WSe_2_ inks display excitonic transitions, around 679 and 623 nm for MoS_2_,^31^ 638 and 526 nm for WS_2_, and 775 and 579 nm for WSe_2_, attributed to the A exciton and B exciton, respectively, consistent with previous reports of LPE and mechanically exfoliated flakes.^30,32,33^ The spectral profiles are consistent with size measurements by AFM (Supplementary Note 2).^34^

### Electrochemical Transistors with TMD Networks

**Figure 2:**
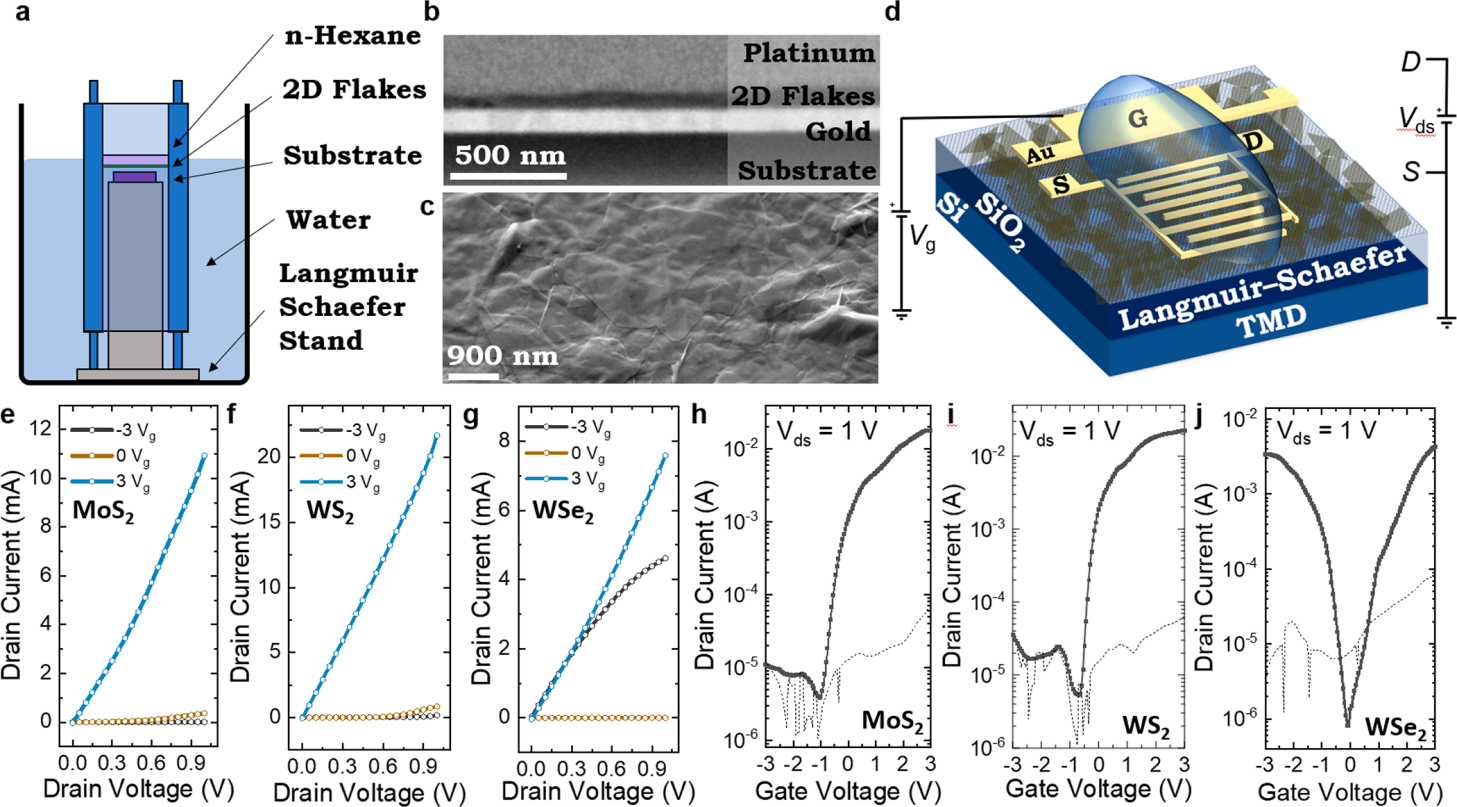
Experimental setup and electrical properties of the Langmuir–Schaefer electrochemical transistor on Si/SiO_2_. (a) Schematic of the Langmuir–Schaefer deposition setup. (b) Cross-sectional SEM of a highly aligned LS network on quartz with *t*_c_ ≈ 60 nm. (c) Scanning electron microscopy image of a LS-deposited MoS_2_ network showing conformal junctions between flakes and folded MoS_2_ flakes (top). Scale bar: 1 μm. (d) Sketch of the Langmuir–Schaefer TMD electrochemical transistor where S is the source and D is the drain (bottom). Output curves of the (e) MoS_2_ transistor, (f) WS_2_ transistor, and (g) WSe_2_ transistor where *I*_d_ is measured as a function of *V*_ds_ (using *V*_g_ ranging from −3 to 3 V with a step change of 3 V). Transfer curves of the (h) MoS_2_, (i) WS_2_, and (j) WSe_2_ transistors where *I*_d_ is measured as a function of the applied *V*_g_ (black curve). For each set of devices, *V*_ds_ = 1 V is used. *I*_g_ is also measured as a function of the applied *V*_g_ (dashed black curve).

We use Langmuir–Schaefer (LS) coating (Figure 2a) to fabricate TMD networks on Si/SiO_2_ wafers with our MoS_2_, WS_2_, and WSe_2_ inks. The interfacial tension at the hexane/deionized water interface creates highly aligned networks after solvent removal with minimal ink wastage of <20 μL (see Methods). As shown in Figure 2b, we used a focused ion beam to cut and polish a cross-section of an MoS_2_ network made by the LS deposition process, which was then imaged using SEM. The cross sections appear largely featureless, implying the film to be near-monolithic. An image analysis^35^ showed this network to contain 2–5% mesopores (i.e., those larger than 5 nm in size—smaller pores are below the resolution of this measurement). This is significantly lower than for poorly aligned, spray-coated nanosheet networks (*P*_net_ ≈ 0.3–0.6),^10,35^ implying LS-deposited films are compact and consist of basal-plane-aligned nanosheets with adjacent sheets lying conformal to each other.^9^ In Figure 2c top-surface SEM imaging of an MoS_2_ network also shows excellent flake alignment in the plane of the film and conformal interflake junctions, implying a low junction resistance and *E*_a_.^9,11^ We also observe folds and wrinkles in the MoS_2_ flakes (also identified with TEM, Supplementary Note 3), implying high flake flexibility which we have previously linked to the formation of conformal junctions.^9^ Gold electrodes (∼100 nm thick) are deposited by evaporation (Temescal FC-2000) through a stainless-steel mask, creating source and drain electrodes with width *W* ≈ 11 mm and channel length *L*_c_ ≈ 50 μm onto networks of MoS_2_, WS_2_, and WSe_2_. A side gate of ∼1.5 mm × 4 mm is also patterned ∼1 mm from the source and drain electrodes (Supplementary Note 4). The devices were then annealed again at 120 °C for 1 h in an inert N_2_ environment. AFM measurements (see Methods) reveal a network thickness (*t*_c_) of ∼25–40 nm (Supplementary Note 3) for our devices. To complete our electrochemical transistor, shown in Figure 2d (bottom), we add the drop-cast ionic liquid 1-ethyl-3-methylimidazolium bis(trifluoromethylsulfonyl)imide (EMIM TFSI) to allow gating of the semiconducting channel.^36^

**Figure 3:**
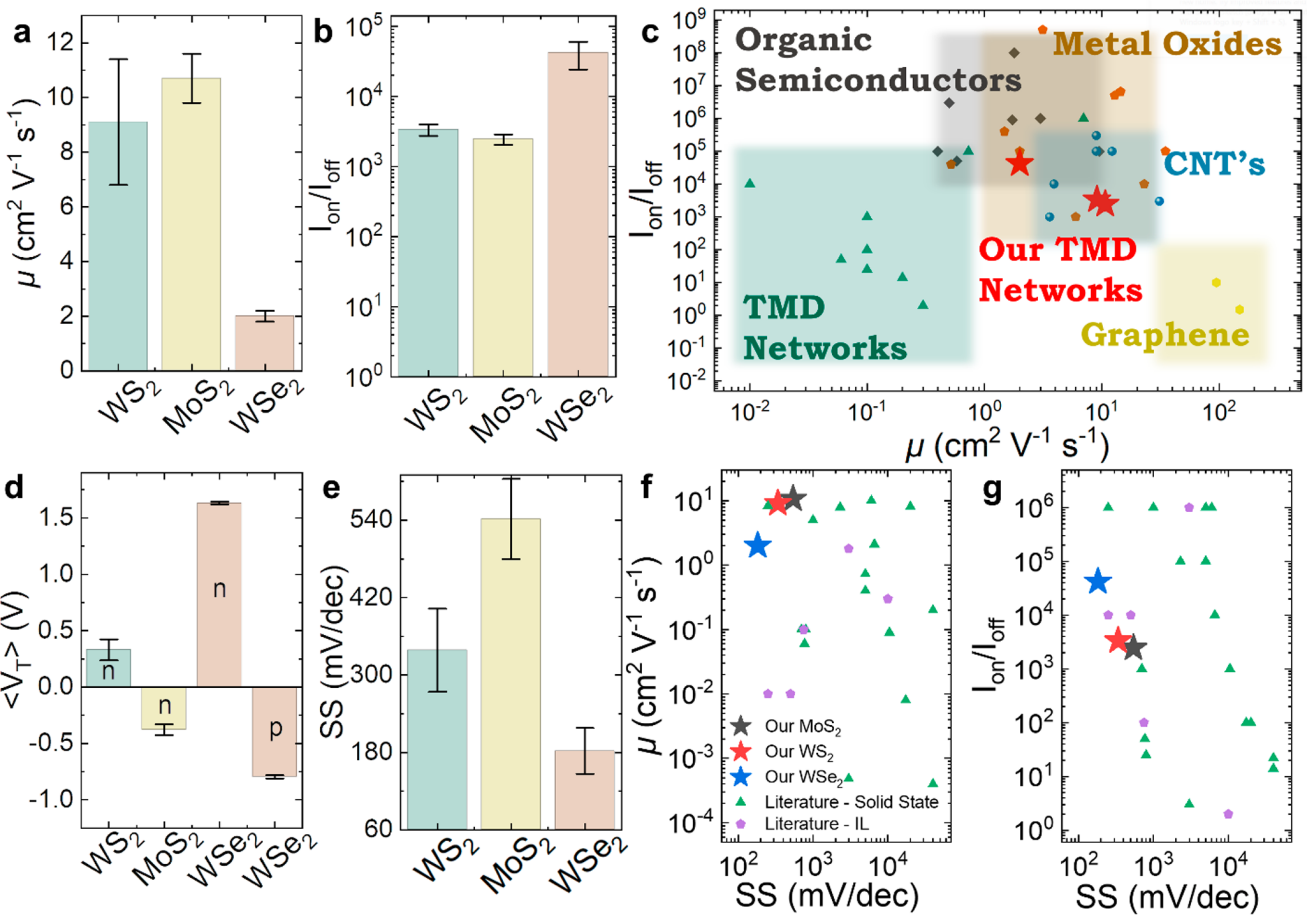
Device performance compared to previous scientific literature. (a) Our transistor μ values for WS_2_, MoS_2_, and WSe_2_. (b) Our *I*_on_/*I*_off_ ratios for WS_2_, MoS_2_, and WSe_2_. (c) Our μ values and *I*_on_/*I*_off_ ratios (red stars) compared to those of other TMD networks (green triangles), organic semiconductors (black squares), metal oxides (orange pentagons), carbon nanotubes (blue circles), and graphene networks (yellow hexagons). (d) The device ⟨*V*_T_⟩ and (e) subthreshold slope for WS_2_, MoS_2_, and WSe_2_. (f) The μ and (g) *I*_on_/*I*_off_ ratio performance plotted as a function of the subthreshold slope for our electrochemical WS_2_, MoS_2_, and WSe_2_ transistors and compared in more detail to TMD networks for solid-state (green triangles) and ionically gated (purple pentagons) devices. Error is calculated by SDOM in this figure.

Next, we characterize the electrochemical transistors using a probe station at atmospheric pressure and temperature and in ambient air. For the MoS_2_, WS_2_, and WSe_2_ (Figure 2e–g) devices, we measure the output characteristics at gate voltages of 3, 0, and −3 V. These curves are consistent with MoS_2_ and WS_2_ being n-type,^3,13^ as the devices switch on at positive *V*_g_ (as seen by a drop in drain current, *I*_d_, to <0.1 mA). The WSe_2_ transistor remains on at −3 *V*_g_ and 3 *V*_g_ and is only off (*I*_d_ < 1 μA) at *V*_g_ ≈ 0 V, confirming ambipolar behavior as expected.^14^ Next, we measure the transfer characteristics using a gate voltage (*V*_g_) window of −3 to 3 V and applying a drain source of *V*_ds_ = 1 V (Figure 2h–j for MoS_2_, WS_2_, and WSe_2_, respectively). We also observed gate leakage (*I*_g_) for each device (dashed black line) attributed to the conductivity of the ionic liquid. The low *I*_g_ indicates the lack of electrochemical reactions with the electrodes. We calculate μ of the transistors from the equation μ = (*L*_c_/*W*)(1/*C*_device_)(*g*_m_/*V*_ds_), where *g*_m_ = ∂*I*_d_/∂*V*_g_ is the transconductance (e.g., measured from the slope of the transfer characteristic from ∼1 to −1 *V*_g_ for n-type MoS_2_/WS_2_ and ∼0 to −1 *V*_g_ for n-type WSe_2_) and *C*_device_ is the device capacitance estimated as ∼3.1 μF cm^–2^ from cyclic voltammetry (see Methods and Supplementary Note 6). The average ambient μ values for MoS_2_, WS_2_, and WSe_2_ are calculated to be μ_MoS_2__ ≈ 10.7 ± 0.9 cm^2^ V^–1^ s^–1^ (*N* = 9), μ_WS_2__ ≈ 9.1 ± 2.3 cm^2^ V^–1^ s^–1^ (*N* = 6), and μ_WSe_2__ ≈ 2.0 ± 0.2 cm^2^ V^–1^ s^–1^ (*N* = 6) (Figure 3a), with *I*_on_/*I*_off_ ≈ (2.6 ± 0.4) × 10^3^, (3.4 ± 0.6) × 10^3^, and (4.2 ± 1.8) × 10^4^ for MoS_2_, WS_2_, and WSe_2_ respectively (Figure 3b). The WSe_2_ p-type μ was also notably high at 1.3 ± 0.2 cm^2^ V^–1^ s^–1^. The best devices had μ_MoS_2__ ≈ 15.1 cm^2^ V^–1^ s^–1^, μ_WS_2__ ≈ 16.3 cm^2^ V^–1^ s^–1^ ,and μ_WSe_2__ ≈ 2.8 cm^2^ V^–1^ s^–1^ with *I*_on_/*I*_off_ ∼ 1.9 × 10^3^, 5 × 10^3^ and 5 × 10^3^ for MoS_2_, WS_2_, and WSe_2_ respectively. We find that the electrical properties are consistent within the array and similar to vacuum-based measurements with no significant decrease in performance when measured in ambient air (Supplementary Note 5). The μ value is orders of magnitude greater than those in previous works on ionic gating of TMD networks (μ ≈ 0.01–0.1 cm^2^ V^–1^ s^–1^)^10,12−14,21^ and either greater than or comparable to those in previous literature (μ ≈ 0.01–8 cm^2^ V^–1^ s^–1^) for solid-state field-effect transistors (FETs).^3,11,15−18,22,37^ We attribute the high μ to both the removal of the residual stabilization agent by centrifugation washing and the use of electrochemical 2D TMDs: their high aspect ratio and LS-induced alignment lead to conformal junctions and thus reduced junction resistance and *E*_a_.^9^ Our devices represent a significant improvement to state-of-the-art solution-processed 2D network literature devices. The transistors are comparable to competing solution-processable technologies developed over the last few decades, such as organic polymers,^38−40^ semiconducting carbon nanotubes (CNTs),^41−43^ graphene^27^ and metal oxides,^44−46^ as shown in Figure 3c. A more comprehensive table can be found in Supplementary Note 7. The n-type behavior of MoS_2_ and WS_2_ could complement the library of high-μ p-type organic polymer materials available for flexible digital electronics, as there is currently a lack of high-μ n-type semiconductors,^47^ essential for CMOS circuits which require both similarly high-μ p-type and n-type transistors.^3^

Minimising the threshold voltage ⟨*V*_T_⟩ is important to reduce the power supply voltage in transistor circuits.^3^ In Figure 3d our MoS_2_ (yellow) and WS_2_ (green) transistors have ⟨*V*_T_⟩ = −0.38 ± 0.05 and 0.33 ± 0.09 V, respectively, demonstrating minimal variation in ⟨*V*_T_⟩ between devices. The n-type ⟨*V*_T_⟩ of the WSe_2_ devices (orange, n) is 1.63 ± 0.01 V, and the p-type ⟨*V*_T_⟩ is −0.80 ± 0.01 V (orange, p), which can likely be attributed to either W or Se vacancies.^48^ In Figure 3e, we calculate the subthreshold slope (SS), defined as the change in gate voltage necessary to change the drain current by one decade. The SS should be minimized to reduce the switching power loss.^49^ We find SS values of 542 ± 62, 339 ± 64 and 182 ± 36 mV/dec for the MoS_2_ (yellow), WS_2_ (green), and WSe_2_ (orange) transistors, respectively. Since SS ∝ 1/ *C*_device_,^50^ our SS is expected to be low (<600 mV/dec) since we use a high *C*_device_ ≈ 3.1 μF cm^–2^, attributed to the ionic liquid EMIM TFSI. Assuming the semiconductor capacitance in our transistor channels is similar, and our *C*_device_ is constant between our electrochemical transistors, the increased MoS_2_ SS (>200 mV/dec) compared to WS_2_ and WSe_2_ could potentially be explained by an increased interface trap capacitance. A higher interface trap density in the MoS_2_ transistors could be attributed to sulfur vacancies or poor flake-to-flake interfaces in our network.^50^ As a further investigation, we examine the dependence of ⟨*L*⟩ on the transistor μ and SS and find μ is maximized and SS is minimized when ⟨*L*⟩ > 1 μm (Supplementary Note 7). Plotting the μ (Figure 3f) and *I*_on_/*I*_off_ values (Figure 3g) as a function of SS, we find that the most optimal devices would be found in each plot’s top left-hand corner. In our case, our MoS_2_, WS_2_, and WSe_2_ transistors have some of the lowest SS and highest μ and *I*_on_/*I*_off_ values recorded for TMD networks (Figure 3f,g), even when compared to other ionically gated networks. A comprehensive list can be found in Supplementary Note 7.

### Optimization of Flake Lateral Size in Electrochemical Transistors

**Figure 4:**
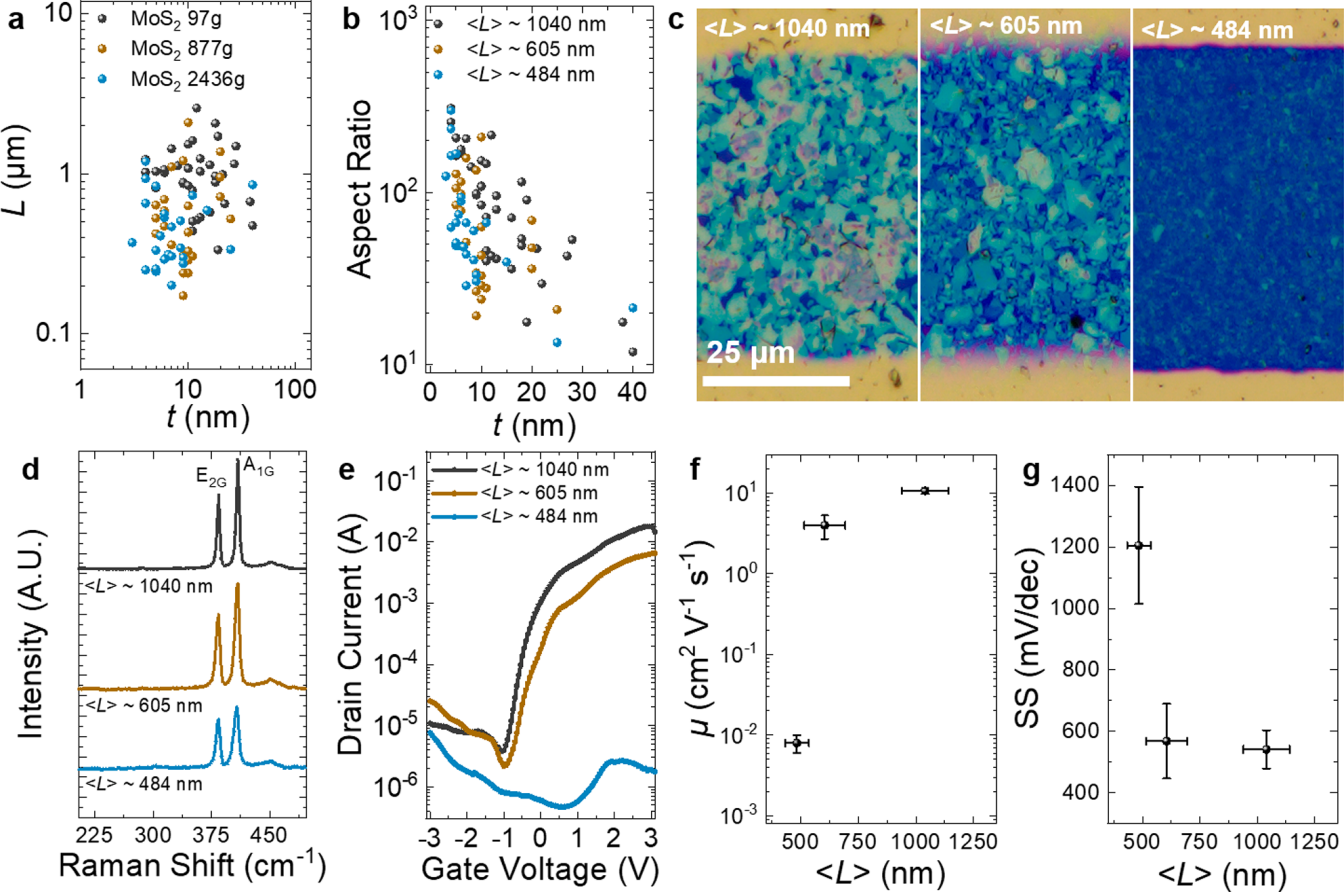
Examination of the effect of ⟨*L*⟩ on MoS_2_ transistor performance. (a, b) Atomic force microscopy statistics of the MoS_2_ flakes. (c) Optical microscopy of the transistor channels with ⟨*L*⟩ from 484 to 1040 nm. (d) Raman spectroscopy of MoS_2_ to determine the defect density in the MoS_2_ flakes. (e) Transfer characteristics of the MoS_2_ flake networks as a function of ⟨*L*⟩. (f) Average μ and (g) average subthreshold slope of the MoS_2_ transistors as a function of ⟨*L*⟩. Error is calculated by the standard deviation of the mean (SDOM) in each case.

As a further investigation, we examine the effect of ⟨*L*⟩ on the transistor performance by making three inks of MoS_2_ of different ⟨*L*⟩ by cascade centrifugation at 97*g*, 877*g* and 2436*g*, respectively, followed by centrifugation washing to remove residual polymer (see Methods). In Figure 4a, AFM estimates ⟨*L*⟩ values of 1040 ± 101, 605 ± 89, and 484 ± 50 nm and ⟨*t*⟩ values of 14 ± 1, 10 ± 1, and 9 ± 2 nm for the 97*g*, 877*g* and 2436*g* MoS_2_ inks, respectively. In each ink, the mean AR was above 70 with many flakes have a value >100 (Figure 4b). A single LS process is used to deposit the MoS_2_ inks on a Si/SiO_2_ substrate, and the previous protocol to pattern the transistors is used (see Methods). We use optical microscopy in the bright field (Figure 4c) to confirm that the TMD flakes cover the channel between the source and drain. The flakes appear to be highly aligned in all cases due to the LS method. As shown in Figure 4d, we use Raman spectroscopy to investigate the defects in each MoS_2_ ink. The Raman spectra look similar, showing the E_2g_ and A_1g_ peaks at 384 and 409 cm^–1^ as the prominent feature.^51^ The intensity ratio of the E_2g_ and longitudinal (LA) mode (*I*(LA)/*I*(E_2g_)) can be used to calculate the defect density in MoS_2_.^51^ The absence of an LA mode peak at ∼330 cm^–1^ is indicative that the MoS_2_ is pristine in the basal plane with a defect density of <0.05 nm^–2^ for each of the three length-selected samples.^51^

In Figure 4e, we characterize the electrochemical MoS_2_ transistors using a probe station at atmospheric pressure and in ambient air. The transfer characteristic (*V*_ds_ = 1 V) shows n-type behavior, which is typical of MoS_2_ for the ⟨*L*⟩ ≈ 1040 nm and ⟨*L*⟩ ≈ 605 nm flake network consistent with previous reports,^3,13,52^ but ambipolar behavior for the ⟨*L*⟩ ≈ 484 nm flakes, possibly due to doping from residual polymer, solvent, or oxygen edge functional groups,^53^ which has been observed previously in MoS_2_ flakes.^54^ The ambient μ values for each MoS_2_ network (Figure 4f) are calculated to be μ_1040_ ≈ 10.7 ± 0.9 cm^2^ V^–1^ s^–1^ (*N* = 9), μ_605_ ≈ 4.0 ± 1.3 cm^2^ V^–1^ s^–1^ (*N* = 3) and μ_484_ ≈ 0.008 ± 0.002 cm^2^ V^–1^ s^–1^ (*N* = 3) with *I*_on_/*I*_off_ ≈ (2.6 ± 0.4) × 10^3^, (3.3 ± 1.9) × 10^3^, and 28 ± 12 for MoS_2_ flakes of ⟨*L*⟩ ≈ 1040, 605, and 484 nm, respectively. The increase in μ with ⟨*L*⟩ suggests the networks to be at least partially junction-limited,^9^ implying further mobility increases are possible. In Figure 4g, we find that the SS decreases with increasing ⟨*L*⟩ from SS ≈ 1205 ± 190 mV/dec at ⟨*L*⟩ ≈ 484 nm to SS ≈ 542 ± 62 mV/dec at ⟨*L*⟩ ≈ 1040 nm. This would suggest a reduced trap capacitance at ⟨*L*⟩ ≈ 1040 nm (since *C*_device_ is constant and the semiconductor capacitance is negligible),^50^ and therefore, we would expect more conformal junctions are being made when ⟨*L*⟩ ≈ 1040 nm. Based on these results, a larger ⟨*L*⟩ should be used when making transistors with TMDs to improve the junctions between flakes and maximize μ and *I*_on_/*I*_off_.

### Flexible WSe_2_ Transistor Arrays

**Figure 5:**
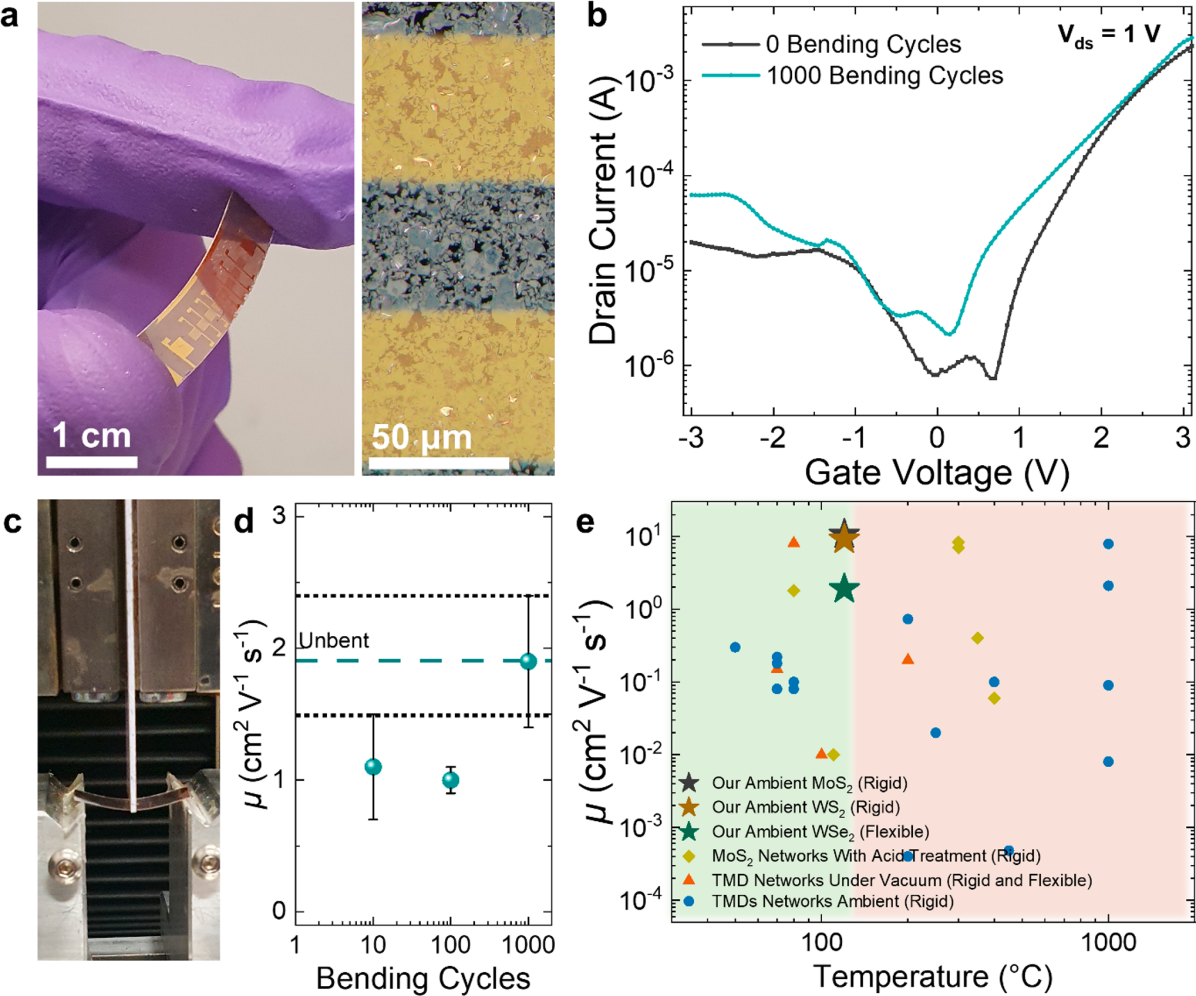
Flexible WSe_2_ transistors on PET. (a) Digital image of the WSe_2_ transistor array on a PET substrate (left) and optical microscopy image of the WSe_2_ channel in bright field (right). (b) Transfer characteristic of the WSe_2_ transistors when unbent (0 cycles) and after 1000 bending cycles using *V*_ds_ = 1 V. (c) Digital image of bending apparatus used for strain testing of devices. (d) Transistor μ as a function of the device bending cycles. The unbent device’s μ is represented by the horizontal cyan dashed line, and the error is represented by the dashed black line. Error is calculated by SDOM. (e) Our flexible WSe_2_ transistor (green star), rigid MoS_2_ (black star) and WS_2_ (brown star) transistors μ as a function of device processing temperature compared to literature values of other solution-processed TMD network transistors made on rigid or flexible substrates. The green box indicates temperatures compatible with flexible substrates, while the red box indicates temperatures incompatible with most flexible substrates (>120 °C).

To investigate our technology’s applicability on a flexible substrate, we undertake a Langmuir–Schaefer deposition of the WSe_2_ ink on PET. We chose WSe_2_, as it had shown the highest *I*_on_/*I*_off_ in the rigid devices. Gold electrodes were evaporated following the protocol established for our previous Si/SiO_2_ devices to make the WSe_2_ transistor array shown in Figure 5a, left (*L*_c_ = 50 μm). A bright field optical microscopy image (Figure 5b, right) shows a uniform deposition of flakes between the source and drain electrodes. The devices are electrically characterized using a probe station in ambient air, and we observe the typical ambipolar behavior expected for WSe_2_ (Figure 5b, black curve). We estimate electron μ ≈ 1.9 ± 0.4 cm^2^ V^–1^ s^–1^ with *I*_on_/*I*_off_ ≈ 2.9 × 10^3^ ± 0.9 × 10^3^, which is similar to the μ_WSe_2__ value obtained for WSe_2_ transistors on Si/SiO_2_ (μ_WSe_2__ ≈ 2.0 ± 0.2 cm^2^ V^–1^ s^–1^, *N* = 6) indicating a successful transfer of the device properties to a flexible substrate despite the higher surface roughness of the PET (*S*_q_ ≈ 18 nm) which would typically result in lower μ.^27^ We then apply a 1% tensile strain to the transistors for 10, 100, and 1000 cycles using the cyclic tensile tester shown in Figure 5c (see Methods) and find that the μ value is maintained (μ ≈ 1.9 ± 0.5 cm^2^ V^–1^ s^–1^, *N* = 6) even after 1000 bending cycles (Figure 5b, cyan curve, and Figure 5d), demonstrating the flexibility of the transistors. In Figure 5e, we plot our transistor’s first-cycle μ performance (green star) against the scientific literature on solution-processed 2D material networks, which are measured in ambient air (blue circles), measured under vacuum (orange triangles), and measured after acid treatment (yellow squares). Most processing methods previously used in the literature are not compatible with flexible substrates, as they typically require annealing temperatures >200 °C or acid treatments and therefore use Si/SiO_2_ or quartz substrates (Supplementary Note 7). Only one work by Kim et al. demonstrated a high-mobility TMD network (MoS_2_) at a low annealing temperature of ∼80 °C, achieving μ ≈ 8.1 cm^2^ V^–1^ s^–1^ and *I*_on_/*I*_off_ ≈ 10^2^ (or μ ≈ 1.8 cm^2^ V^–1^ s^–1^ and *I*_on_/*I*_off_ ≈ 10^6^ on acid treatment) but with a high SS ≈ 3000 mV/dec. However, it was not tested on a flexible substrate but was assembled on Si/SiO_2_ and was measured under vacuum.^17^ To our knowledge, the only two other works that have demonstrated transistors made on PET from a network of WSe_2_ flakes achieved μ ≈ 0.1 cm^2^ V^–1^ s^–1^ and *I*_on_/*I*_off_ ≈ 10^2^–10^3^ and were both measured under vacuum rather than ambient air.^10,14^ Therefore, our WSe_2_ transistors represent a 1 order of magnitude improvement in μ on flexible PET substrates while demonstrating that their performance can be maintained even after excessive straining (1000 cycles at 1% strain). We also note that, to our knowledge, our devices are the only example of flexible high-mobility (μ > 1 cm^2^ V^–1^ s^–1^) TMD networks without using acid treatments, high-temperature annealing, or measurement under vacuum/encapsulation. It is also likely that our MoS_2_ (black star) and WS_2_ (brown star) transistors would also work on a flexible substrate, given that WSe_2_ was successfully transferred without a decrease in μ and the MoS_2_ and WS_2_ follow the same manufacturing and deposition protocol.

## Conclusions

We discover that WS_2_ and WSe_2_ can achieve high-μ transistors with n-type and ambipolar behavior, respectively, to add to the library of high-μ 2D solution-processed materials which will be required in future devices and circuits that need materials with complementary behavior. We successfully utilized Langmuir–Schaefer deposition to minimize ink waste (<20 μL) and improve the network stacking and alignment, enabling ambient air electrochemical transistors with μ ≈ 2–16 cm^2^ V^–1^ s^–1^, *I*_on_/*I*_off_ ≈ 10^3^–10^4^, ⟨*V*_T_⟩ ≈ 0.3–1.6 V, and SS ≈ 182–542 mV/dec representing a 1 order of magnitude increase in μ and SS for state-of-the-art electrochemical transistors with 2D WS_2_ and WSe_2_ flake networks. The performance was comparable to that of solution-processed solid-state 2D network FETs, CNTs, metal oxides, and organic polymers but without high-temperature annealing (>120 °C), acid treatments, and vacuum measurements, improving the commercialization potential of our solution-processed transistors. We also found that the transistor μ is maximized and SS is minimized when ⟨*L*⟩ > 1 μm. As a final demonstration, we created flexible WSe_2_ transistors on PET that did not degrade in performance for at least 1000 bending cycles at 1% strain, showing a 1 order of magnitude improvement in μ for solution processed 2D flakes on a flexible substrate.

## Methods

### Electrochemical Exfoliation of 2D Crystals

An electrochemical cell with two electrodes is used to intercalate WS_2_, WSe_2_, and MoS_2_ (HQ graphene) crystals. A thin piece (0.1 × 1 × 1 mm) of a crystal is used as the cathode, while a platinum foil (Alfa Aesar) is used as the anode. Copper crocodile clips are used to hold the electrodes in place. For the electrolyte, tetrapropylammonium (TPA) bromide (Sigma-Aldrich, 5 mg/mL) is added to propylene carbonate (∼50 mL). A voltage of 8 V is applied for 30 min between the electrodes to intercalate the 2D crystal with TPA^+^ cations. The 2D crystal expands in each case to greater than twice its original volume, indicating the successful intercalation of the crystal. After intercalation, the 2D crystal is emersed in IPA overnight to dissolve and remove any residual bromide ions (Br ^–^) on the crystal.

### Ink Formulation with 2D Crystals

The 2D crystal is then bath-sonicated (Fisherbrand 112xx series) in 1 mg/mL poly(vinylpyrrolidone) (PVP, molecular weight ∼40000) in dimethylformamide (DMF) for 5 min followed by centrifugation (Hettich Mikro 220, 1195-A, radius 87 mm) at 500 rpm (24*g*) for 20 min to remove unexfoliated crystals. The dispersion is size-selected by centrifuging the supernatant (top 90%) at 1000 rpm (97*g*) for 1 h and collecting the sediment. Unfortunately, attempts to disperse the 2D crystal directly in DMF without PVP were unsuccessful, as the initial centrifugation step (24*g*) resulted in complete sedimentation of the unexfoliated and exfoliated crystals. Therefore, size selection and removal of bulk unexfoliated crystals would not be possible. To remove the PVP, the 97*g* sediment was diluted with 2 mL of DMF and centrifuged at 10k rpm (9744*g*) for 1 h. The process was repeated twice, and the sediment was collected each time. A third washing step was used to remove residual DMF, which involved diluting the sediment in IPA (0.5 mL) and subsequently centrifuging at 10k rpm (9744*g*) and collecting the sediment. The sediment is redispersed in IPA (∼0.5 mL, concentration ∼2.5 mg/mL) to make the 97*g* dispersion used in the study respectively for each 2D crystal. We use IPA, as it is a low-boiling-point solvent (∼82.5 °C) that can evaporate quickly after Langmuir–Schaefer deposition. For the ⟨*L*⟩ study, the supernatant of the 1000 rpm (97*g*) MoS_2_ dispersion is centrifuged at 3000 rpm (877*g*) and then 5000 rpm (2436*g*). The sediment of the 877*g* and 2436*g* MoS_2_ dispersions follows the washing protocol previously described and is then redispersed in IPA to make the 877*g* MoS_2_ ink and 2436*g* MoS_2_ ink, respectively.

### Network Formation by Langmuir–Schaefer and Transistor Electrode Fabrication

The Langmuir–Schaefer setup involves a Teflon stand (10 cm long) where a Si/SiO_2_ chip (2 × 2 cm) is placed on top (root-mean-square roughness *S*_q_ ≈ 0.1 nm, 300 nm oxide thickness). The stand is placed in a beaker (about 100 mL) of deionized water. About 20 mL of distilled hexane is drop-cast onto the surface of the deionized water to create a water/hexane interface, under which the Si/SiO_2_ chip is submerged. The MoS_2_, WS_2_, and WSe_2_ inks are drop-cast (∼140 μL) onto the surface of the hexane until no gaps in the interface could be seen. The Teflon stand and Si/SiO_2_ are then carefully extruded through the 2D crystal layer to coat the surface of the Si/SiO_2_ with the TMD network; ∼20 μL of material is lost at the edges of the Si/SiO_2_. The TMD networks are left in a fume hood to dry in ambient air for ∼6 h. Next, we anneal the TMD networks at 120 °C for 1 h on a hot plate in an N_2_ glovebox (Jacomex GP campus) to remove residual solvent and improve the adhesion of the 2D flakes to the Si/SiO_2_ substrate. The process is repeated to build the second layer of the network. Two depositions are undertaken for the WS_2_ and WSe_2_ networks, while one deposition is used for MoS_2_. Gold electrodes (∼100 nm thick) are deposited by evaporation (FC-2000 Temescal Evaporator) through a stainless steel mask (50 μm thick) which is laser cut (Laser Micromachining ltd). The gold defines the channel dimensions of *W* = 11000 μm and *L*_c_ = 50 μm. The Au evaporation also defines our gate electrode, which is placed ∼1 mm from the source and drain electrodes and is ∼1.5 × 4 mm in size. The WS_2_, WSe_2_, and MoS_2_ devices (each on individual chips) are then annealed on a hot plate at 120 °C for 1 h in an inert N_2_ environment (Jacomex GP campus). The same protocol is used for our WSe_2_ devices on a flexible substrate, replacing Si/SiO_2_ with PET (Novele, Novacentrix).

### Transmission Electron Microscopy

TEM is performed using a JEOL 2100 instrument. The TEM grids are prepared by LS deposition of the MoS_2_ ink (1 layer, see Langmuir–Schaefer methods for protocol) on lacey-carbon grids followed by room-temperature drying for ∼6 h. The TEM imaging is performed at an accelerating voltage of 200 kV using a beam current of 105 μA.

### Scanning Electron Microscopy

SEM is performed with a Carl Zeiss Ultra SEM operating at 4 kV with a 30 μm aperture. Images are acquired using the secondary electron detector, and the samples are not coated prior to imaging. The sample substrate is a 300 nm SiO_2_/Si wafer.

### FIB-SEM Cross-Section Imaging

FIB-SEM microscopy is carried out using a dual-beam Carl Zeiss Auriga focused ion beam system. Network cross sections are milled using a 30 kV:600 pA beam. All images are captured at a working distance of 5 mm with a 2 kV accelerating voltage and aperture size of 30 μm. The network porosity is measured by segmenting network cross sections into their pore and nanosheet contributions using trainable WEKA segmentation.^55^ The porosity is calculated by dividing the number of pixels classified as “pore” by the numerical sum of the “pore” and “nanosheet” pixels in each cross-section. This technique can identify pores larger than 5 nm × 5 nm in cross-sectional area.

### *I*–*V* Probe Station Measurements

To control the injection of ions into our semiconducting channel, we use the ionic liquid 1-ethyl-3-methylimidazolium bis(trifluoromethylsulfonyl)imide (EMIM, Sigma-Aldrich). The ionic liquid is first heated under vacuum at 100 °C for 6 h to remove any absorbed water. A drop of EMIM is then pipetted onto the transistors so that the gate, source, and drain electrodes are covered with ionic liquid. The devices are left under vacuum (∼1.6 × 10^–4^ mbar) in a Janis Probe Station overnight (12 h) to remove residual water further. Before undertaking measurements, the devices are brought back to atmospheric pressure. To undertake electrical characterization, devices are contacted using gold-coated probes connected to a Keithley 2612A dual-channel source measuring unit. A gate voltage window of −3 to 3 V is used for transfer characteristics with a scan rate of 50 mV/s and *V*_ds_ = 1 V for all devices.

### Cyclic Voltammetry

A Gamry Reference 600 Potentiostat is used to undertake cyclic voltammetry measurements. The capacitance was extracted from area enclosed by the CV curves. We estimate the *C*_device_ value to be ∼3.1 μF cm^–2^ for our MoS_2_ and WS_2_ devices and ∼4.9 μF cm^–2^ for our WSe_2_ devices. *C*_device_ is also estimated as ∼1.2 μF cm^–2^ for ⟨*L*⟩ ≈ 484 nm MoS_2_ devices (see Supplementary Note 6 for further information).

### Optical Microscopy

An optical microscope (Olympus DSX1000 digital microscope) is used to image deposited droplets in bright field mode. The images are acquired at a ×50 magnification. For the device’s imaging, a single 70 × 70 μm image is not sufficient to observe the entire device. Therefore, an area of 7 × 7 images is sequentially taken and stitched together with a 10% overlap in live panorama mode.

### Atomic Force Microscopy

A Bruker Multimode 8 microscope is used to undertake AFM and analyze the thickness and lateral size of the flakes. The WS_2_, MoS_2_, and WSe_2_ inks are drop-cast onto Si/SiO_2_ after dilution in IPA by a factor of 1:100. The samples are then annealed at 120 °C for 15 min to remove residual solvent. The samples are scanned using OLTESPA R3 cantilevers in ScanAsyst mode, and ∼35–50 flakes are counted to determine the statistics. The lateral size is calculated as the square root of the flake length times the flake width.

### Raman Spectroscopy

Inks of MoS_2_, WS_2_, and WSe_2_ are drop-cast onto an Si/SiO_2_ substrate and annealed at 120 °C. The Raman spectra of the drop cast networks are acquired with a Horiba Jobin Yvon Labram HR800 Raman system at 532 nm with a 10× objective and incident power of ∼1 mW to minimize possible thermal damage.

### Optical Absorption Spectroscopy

The extinction spectra are obtained using a PerkinElmer Lambda 1050 spectrometer at a step of 1 nm with a 10 mm optical length cuvette (quartz cuvette). The absorption spectra are obtained by using an integrating sphere. The slit width is 2 nm.

### Transistor Three-Point-Flexural Tests

A zwickiLine (ZwickRoell) three-point flexure testing system is used to conduct bending testing on the transistors. The strain applied (ε) to our transistor can be calculated using the equation ε = 6*dD*_f_/*L*_s_^2^, where *d* is the PET thickness (170 nm), *L*_s_ is the support span (16 mm), and *D*_f_ is the maximum deflection of the center of the beam. In all cases, we apply a strain of 1%. To obtain an average in our mobility estimate we use *N* = 6, 7, 6, and 6 transistors for 0, 10, 100, and 1000 bending cycles, respectively.

## Data Availability

The authors declare that the data supporting the findings of this study are available within the paper and its Supporting Information files. Data are also available from the corresponding author upon reasonable request.

## Supporting Information Available

Aspect ratio comparison between shear mixing and electrochemical inks, UV–vis of the TMD flakes, imaging with SEM, TEM, and AFM of the flake-to-flake junctions in the networks, optical microscopy of the devices, electrical characteristics of the device arrays including examination of device gate leakage and hysteresis, cyclic voltammetry of the networks, and a literature review of TMD transistors with solution-processed networks (PDF)
