# Supplementary material for: High-Mobility Flexible Transistors with Low-Temperature Solution-Processed Tungsten Dichalcogenides
Source: ACS Nano. 2023 Jan 31;17(3):2912–22. doi: 10.1021/acsnano.2c11319 (PMC9933598; doi:10.1021/acsnano.2c11319)
Supplement: Supplementary file 1 — nn2c11319_si_001.pdf [file nn2c11319_si_001.pdf]

Supporting Information

High Mobility Flexible Transistors with Low Temperature Solution Processed Tungsten Dichalcogenides

Tian Carey[1], Oran Cassidy[1], Kevin Synnatschke[1], Eoin Caffrey[1], James Garcia[1], Shixin Liu[1], Harneet Kaur[1], Adam G. Kelly[1], Jose Munuera[1], Cian Gabbett[1], Domhnall O'Suilleabhain[1], Jonathan N. Coleman[2*]*

[1]CRANN and AMBER Research Centres, Trinity College Dublin, Dublin, D02 E8C0, Ireland

*Correspondence and requests for materials should be addressed to T.C. (tian.carey@cantab.net) and J.C. (colemaj@tcd.ie)

Supplementary Note 1

Aspect ratio comparison between shear mixing and electrochemical inks

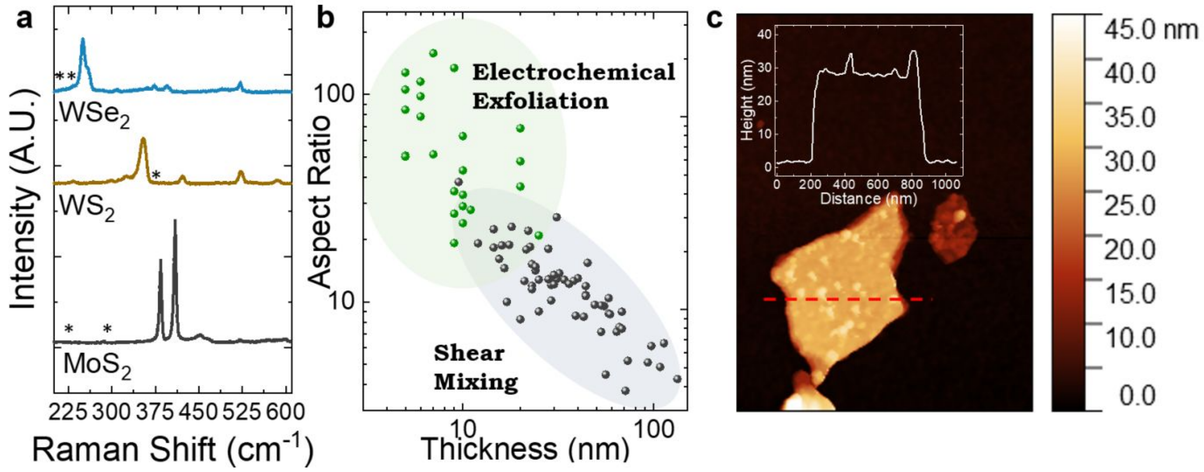

Supplementary Figure 1: Raman Spectroscopy and comparing EE to LPE MoS_2 inks. a Chemical analysis by Raman spectroscopy of the TMD flakes after exfoliation **b** AFM statistics of the electrochemically exfoliated and high-shear mixed MoS_2 ink (97g) AR as a function of *t*. **c** AFM micrograph of a typical shear mixed MoS_2 flake.

Supplementary Figure 1a depicts the spectra of the MoS_2 (black), WS_2 (brown) and WSe_2 (blue) flakes after drop casting and annealing at 120 °C on Si/SiO_2. The black MoS_2 spectrum (Supplementary Figure 1a) shows the typical E_{2g} and A_{1g} peaks at 384 cm^{-1} and 409 cm^{-1}, respectively.[1, 2] The Raman spectrum of the WS_2 (Supplementary Figure 1a, brown) also matches the previous literature reports for few-layer WS_2, having an A_{1g} peak (~ 421 cm^{-1}) and an overlapping 2LA and E_{2d} peak at 354 cm^{-1}.[3] The peak at 521 cm^{-1} is attributed to the Si/SiO_2 substrate. The WSe_2 (Supplementary Figure 1a, blue) has a peak at (~ 250 cm^{-1}) attributed to the A_{1g} and E_{2g} Raman modes, indicating the formation of few-layer flakes.[4, 5] For the MoS_2, WS_2 and WSe_2, the J_2 and J_3 vibrational modes attributed to the metallic 1T phase are not observed.[6-9] The absent 1T phase peaks (marked by an asterisk to show their location) would be located at 224 and 289 cm^{-1} for MoS_2, 385 cm^{-1} for WS_2 and 218 and 236 cm^{-1} for WSe_2.

To compare the flakes of electrochemical exfoliation to high-shear mixing, we make two MoS_2 inks. First, the electrochemically exfoliated ink is made as described in the main text. Then, for the shear mixed MoS_2 ink, we use MoS_2 powder (Alfa Aesar, 10 mg/ml) with 1 mg/ml PVP as a stabilisation agent in DMF and shear mix with a 4-blade rotor (Silverson Model L5M) for 8 hours (8000 rpm). The rotor had a rotor-stator gap of 300 μm and a rotor diameter of 31.1 mm. The dispersion was centrifuged at 500 rpm (24g) for 20 minutes following shear mixing to remove bulk MoS_2. The dispersion is then size selected by centrifuging the supernatant (top 90 %) at 1000 rpm (97g) for 1 hour. Finally, the sediment is collected and redispersed in IPA to make the shear mixed MoS_2 ink. For a fair comparison between the shear mixed MoS_2 ink and electrochemically exfoliated $MoS_{2,}$ the starting MoS_2 and PVP concentration were the same.

Furthermore, the centrifugation conditions and solvent used were the same. The two inks were drop-cast on separate Si/SiO_2 chips after dilution in IPA by a factor of 1:100. The samples were then annealed at 120 °C for 15 min to remove residual solvent. A Bruker Multimode 8 microscope was used to undertake AFM and analyse the thickness and lateral size of the flakes using OLTESPA R3 cantilevers in ScanAsyst mode. In supplementary figure 1b, we observe an aspect ratio of the shear mixed flakes between 4 – 38, with the majority having an aspect ratio of ~10. Supplementary figure 1c shows a typical flake found in the shear-mixed MoS_2 ink with an apparent thickness of ~ 30nm. Conversely, the aspect ratio of the electrochemically exfoliated flake could be >100. Therefore it is more likely that the electrochemically exfoliated flakes will make more conformal (flake-to-flake) junctions when aligned in a network.[10]

Supplementary Note 2

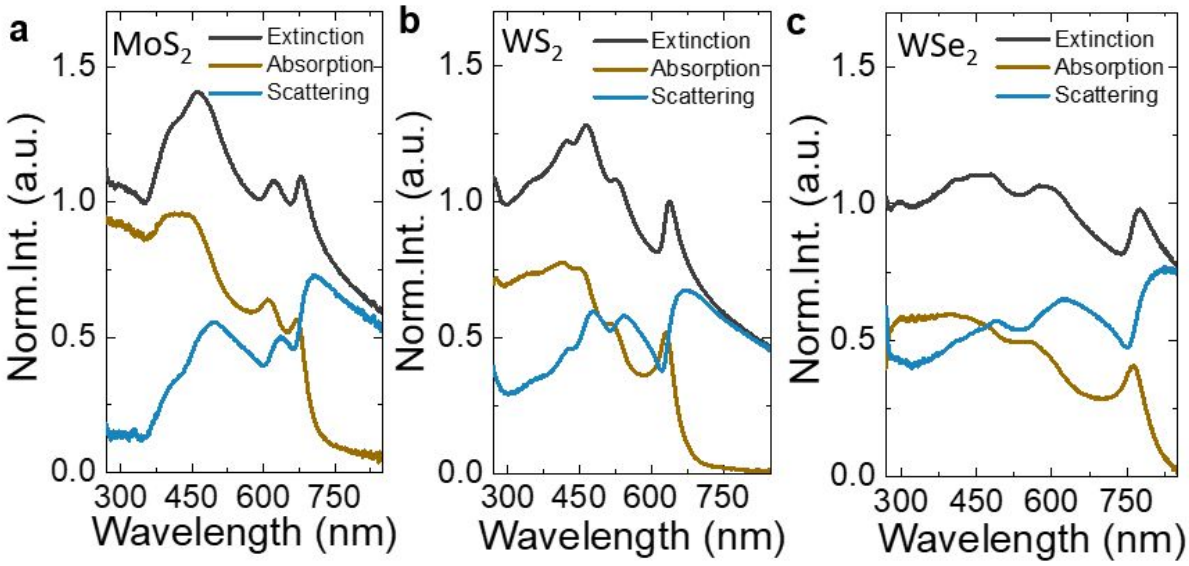

Supplementary Figure 2: **UV-vis of TMD flakes. a,b,c** Normalised optical characterisation of the TMD inks by UV-vis showing the extinction (black), absorption (brown) and scattering (blue) components as a function of wavelength shone through the ink.

In Supplementary Figure 2a, 2b and 2c UV-visible optical absorption spectra of the MoS_2, WS_2 and WSe_2 inks are taken with an integrating sphere to isolate the extinction, absorption and scattering components of the TMD inks.[11] The scattering component of the TMD inks is ~50% of the extinction spectra intensity and would suggest that the flakes have a large *L*. The ratio of the extinction at the B exciton peak (Ext_B) to the local minimum at 345 nm (Ext_{345}) in the MoS_2 spectra (supplementary figure 2a, black curve) can be used to measure *L*.[11] The $Ext_B/Ext_{345} > 1$ indicates that the flakes are large with $L > 400$ nm.[11] Similarly, using the ratio of the extinction at the A exciton peak (Ext_A) to the local minimum (Ext_A/Ext_{295}), the *L* of WS_2 can be determined.[11] We find $Ext_A/Ext_{295} > 1$ indicating $L > 400$ nm in agreement with our AFM statistics from the main text.[11, 12]

Supplementary Note 3

Microscopy of MoS_2 flakes and networks

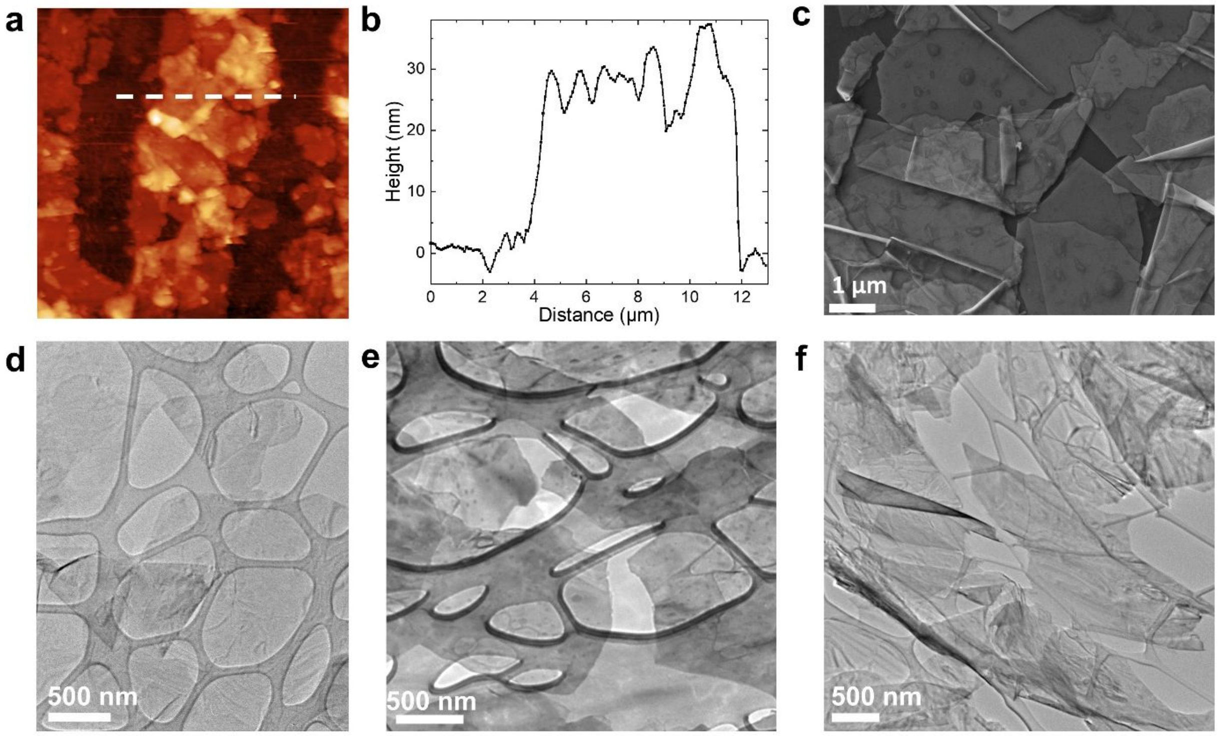

Supplementary Figure 3: **Imaging the flake-to-flake junctions in the LS deposited MoS_2 network.** **a** AFM of the MoS_2 network after LS deposition of the 97g MoS_2 ink. **b** AFM micrograph showing a network thickness of ~ 25 nm. **c** Scanning electron microscopy image of the 97g MoS_2 ink showing ranging from L ~ 1 µm to up to 5 µm for the 97g MoS_2 ink. **d,e,f** Transmission electron microscopy images of the 97g MoS_2 ink deposited by LS on showing flakes with L > 1 µm. Folds and wrinkles in the MoS_2 flakes are also observable.

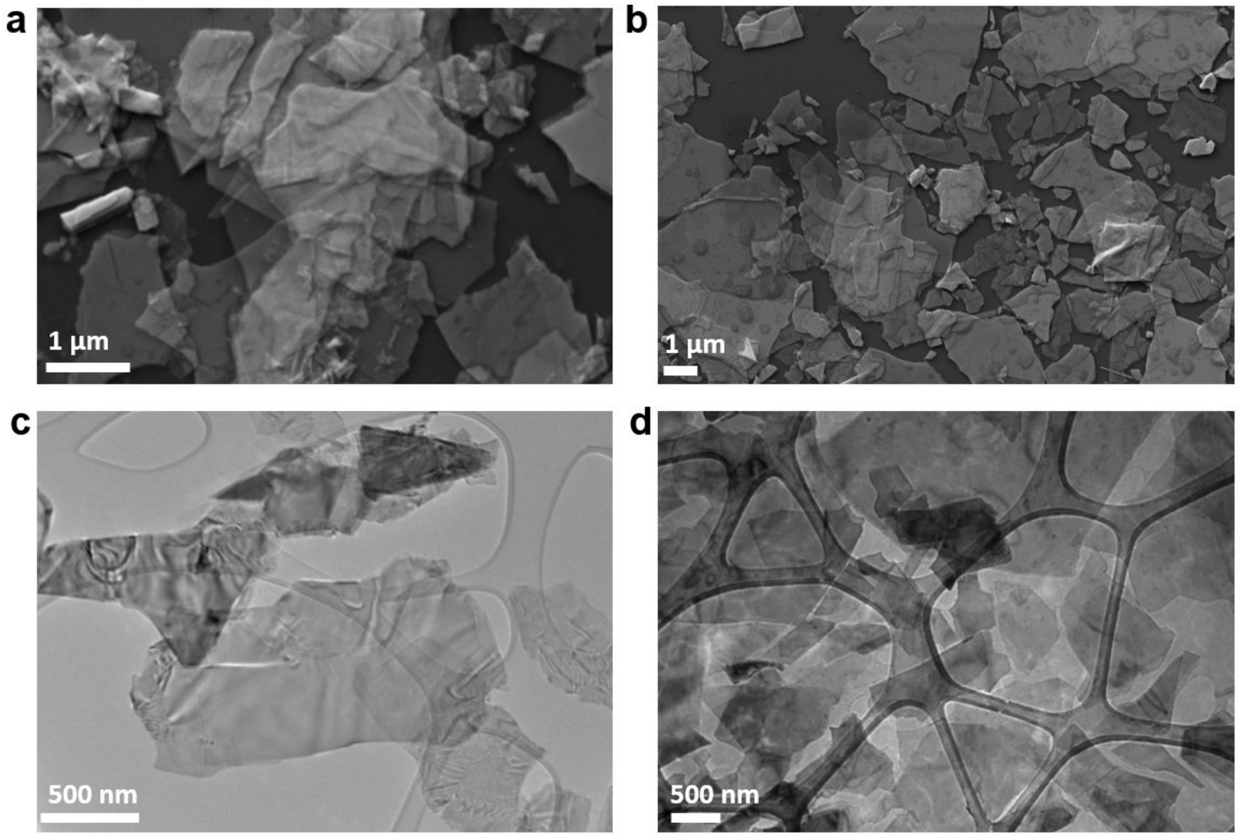

Supplementary Figure 4: **SEM and TEM of WS_2 and WSe_2 networks. a** SEM image of the WS_2 LS network and **b** WSe_2 LS network showing conformal junctions between the flakes. **c, d** Transmission electron microscopy images of the 97g WSe_2 ink deposited by LS on showing flakes with L > 1 µm.

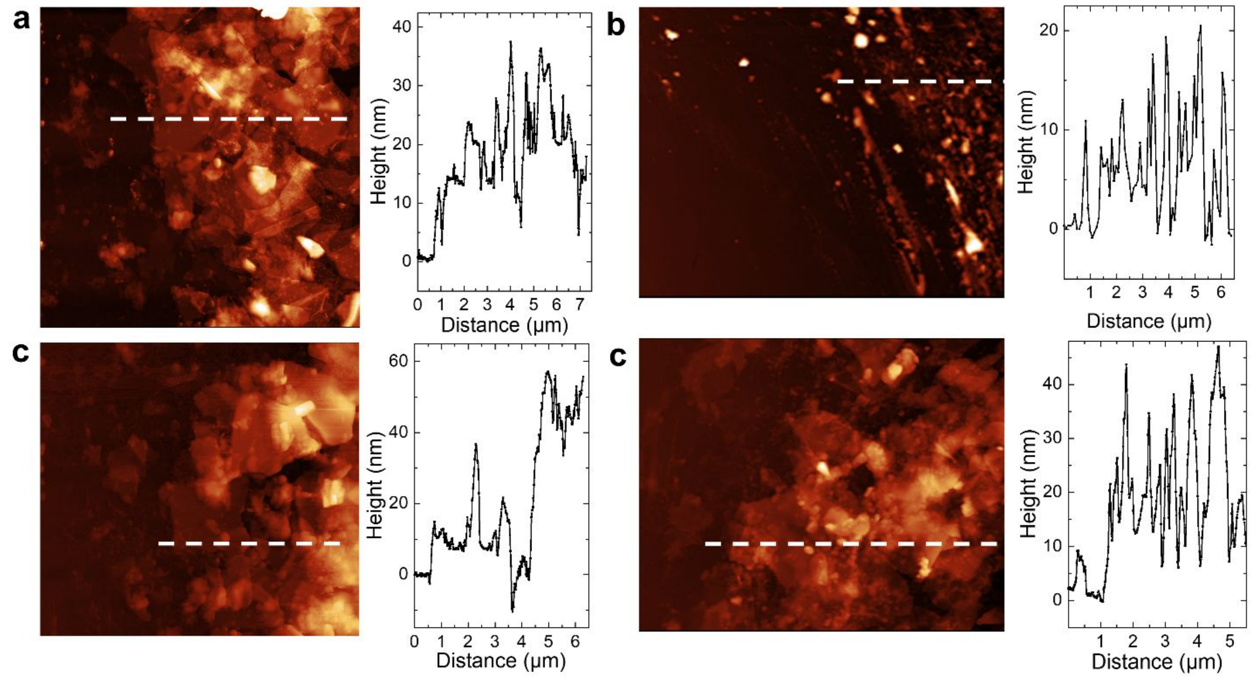

Supplementary Figure 5: **Atomic Force Microscopy of TMD networks. a** AFM micrograph and height profile of the WS_2 network after LS deposition of the WS_2 ink. **b** AFM micrograph and height profile of the MoS_2 network after LS deposition of the 2436g MoS_2 ink. **c** AFM micrograph and height profile of the WSe_2 network after LS deposition of the WSe_2 ink. **d** AFM micrograph and height profile of the MoS_2 network after LS deposition of the 877g MoS_2 ink.

A Bruker Multimode 8 microscope was used to undertake AFM and analyse the thickness the LS deposited films. In Supplementary Figure 3a the MoS_2 network was scanned using a OLTESPA R3 cantilever in ScanAsyst mode. We observe a MoS_2 network thickness of ~ 25 nm (Supplementary Figure 3b). In Supplementary Figure 3c we undertake SEM to image the flake-to-flake junctions for our <*L*> ~ 1040 nm MoS_2 ink on SiO_2/Si wafer. A Carl Zeiss Ultra SEM operating at 4 kV with a 30 μm aperture was used to acquire images using the secondary electron detector, and the samples were not coated before imaging. We observe wrinkles and folds in the <*L*> ~ 1040 nm MoS_2 ink, suggesting the flakes are highly flexible and beneficial for making conformal junctions. In Supplementary Figures 3d, 3e and 3f, transmission electron microscopy was performed using the JEOL 2100 instrument. The TEM grids were prepared by LS deposition of the MoS_2 ink (1 layer, see Langmuir-Schaefer methods for protocol) on lacey carbon grids followed by room temperature drying for ~ 6 hours. The TEM imaging was performed at an accelerating voltage of 200 kV using a beam current 105 μA. In Supplementary Figure 3f, folds in the MoS_2 flakes can be observed, matching our observation from SEM. In Supplementary Figure 4 we show that the conformal networks of flakes are also seen in TEM and SEM for the WS_2 and WSe_2 networks. In Supplementary Figure 5, we undertake further AFM to characterise the thickness of the other WS_2, WSe_2 and MoS_2 networks used in the main text. AFM micrograph reveals a network thickness of ~ 25 nm for WS_2 (Supplementary Figure 5a) and ~ 40 nm for WSe_2 (Supplementary Figure 5c). We also determine the thickness of our

MoS_2 networks used for the <*L*> dependence study in Figure 4 (main text). We find a thickness of ~ 25 nm for the <*L*> ~ 605 nm flake network of MoS_2 and a thickness of ~ 10 nm for the <*L*> ~ 484 nm flake network.

Supplementary Note 4

Optical Microscopy of Devices

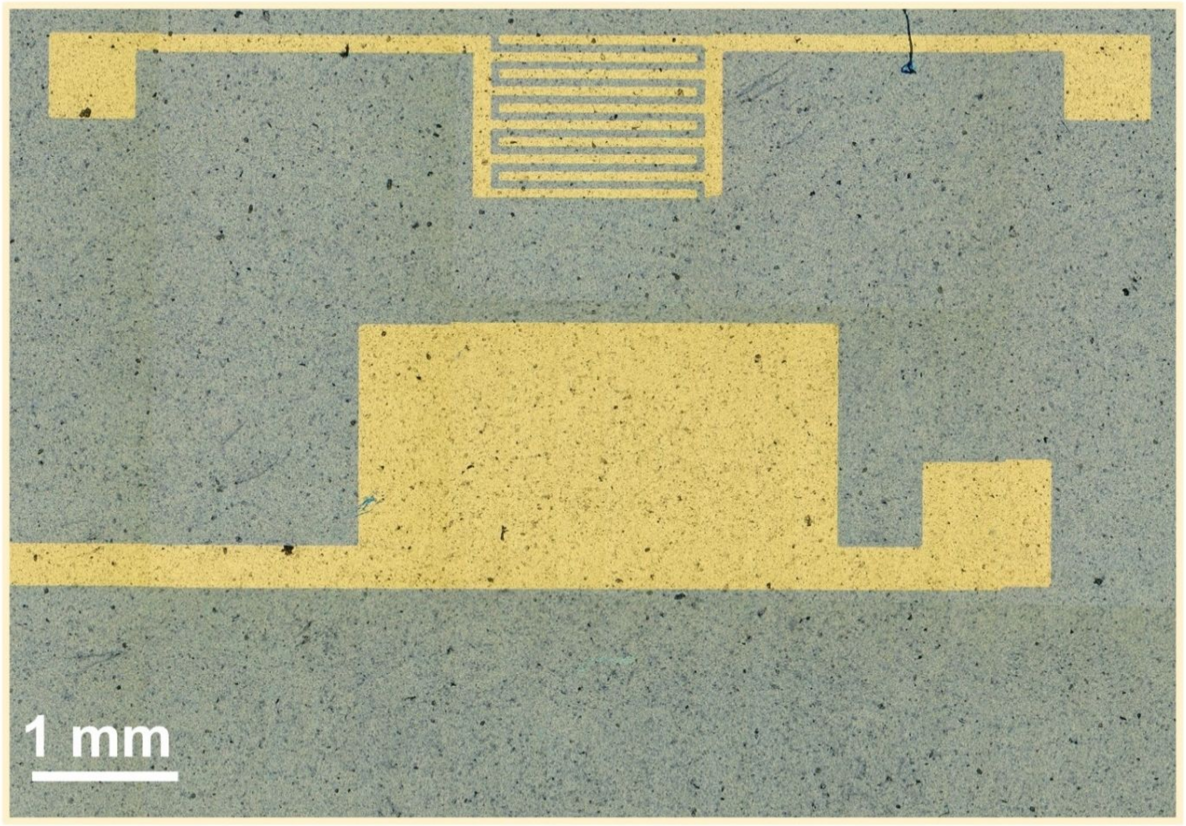

Supplementary Figure 6: **Imaging of WSe_2 LS device.** Optical microscopy in the bright field of the WSe_2 electrochemical transistor on Si/SiO_2.

Supplementary Note 5

Uniformity of electrical characteristics

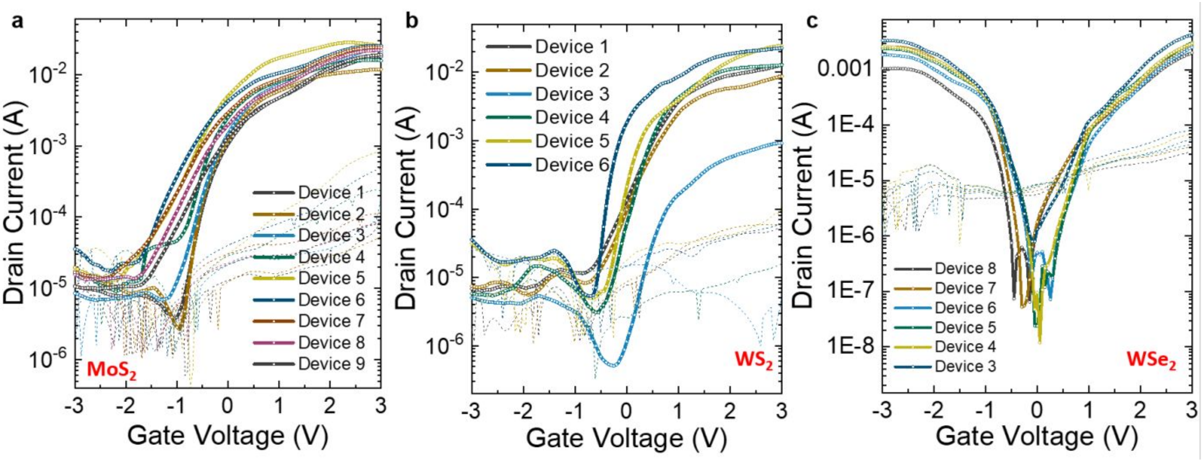

Supplementary Figure 7: **Investigating the uniformity of electrical characteristics. a,b,c** Transfer characteristic of the MoS_2, WS_2 and WSe_2 electrochemical transistors in air ambient demonstrating the n-type behaviour of MoS_2, WS_2 and ambipolar behaviour of WSe_2 with similar electrical characteristics in each case. V_{ds} is set to 1 V for all devices. The dashed line represents the gate leakage I_g for each device.

Examination of device gate leakage and hysteresis

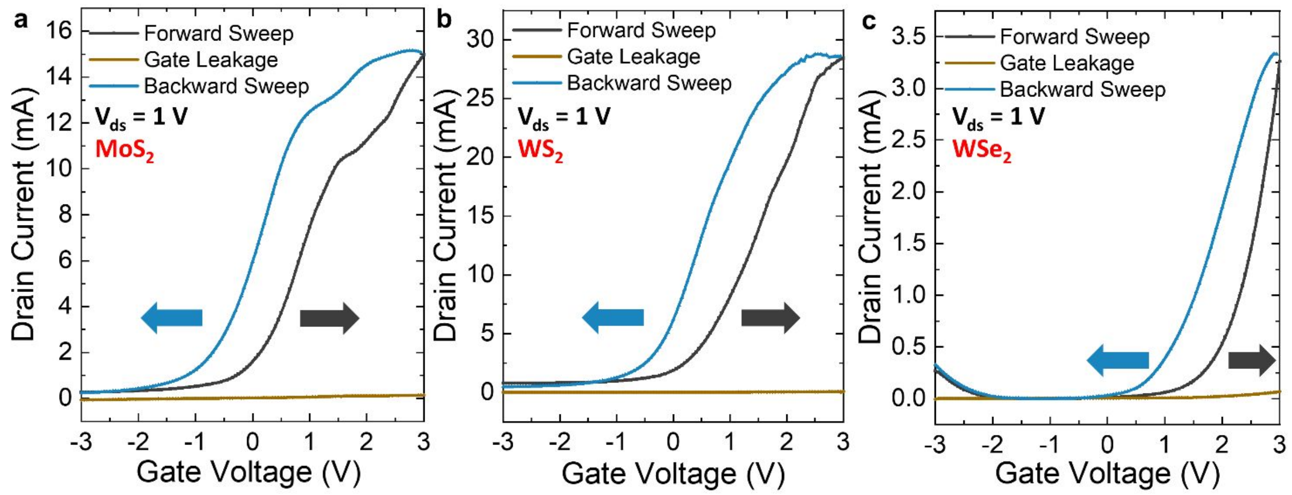

Supplementary Figure 8: **Investigation of device gate leakage and hysteresis. a,b,c** Transfer characteristic of the MoS_2, WS_2 and WSe_2 electrochemical transistors under vacuum with I_d shown as the black and blue curve and the I_g shown as the brown curve. In each case the black curve the black curve is the forward sweep and the blue curve is the backward sweep.

Hysteresis in electrochemical transistors is typically caused by low ionic mobility.[13] In our transfer curves, we note minimal hysteresis after conducting a forward (from -3 V_g to 3 V_g) and backward V_g sweep, probably due to the low network thickness.[14] In addition, the presence of defects/impurities in the semiconductor and ionic liquid could inhibit ion movement and be the cause of the hysteresis.

Supplementary Note 6

Cyclic Voltammetry of MoS_2 network

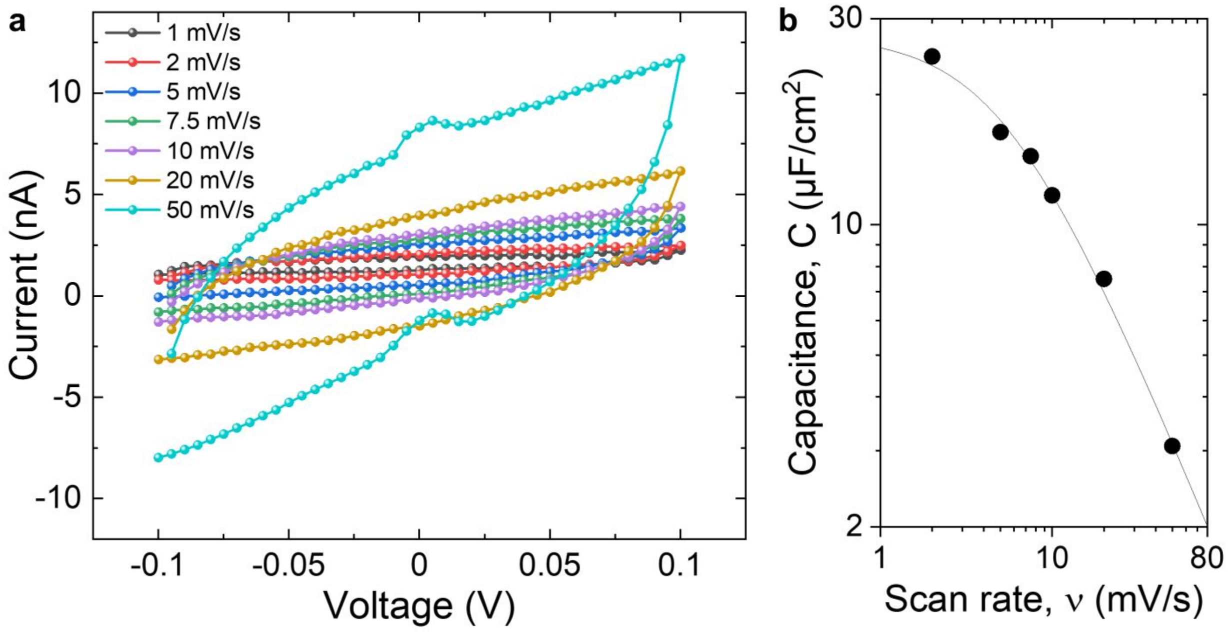

Supplementary Figure 9: **Electrical characterisation of the ionic liquid. a** Cyclic voltammogram measured for MoS_2 deposited on Si/SiO_2 using EMIM TFSI as the electrolyte. **b** Electrochemical transistor capacitance as a function of scan rate.

A Gamry Reference 600 Potentiostat was used to perform cyclic voltammetry measurements. The ionic liquid was first heated under a vacuum at 120 °C to remove any absorbed water, as electrolytes are typically hygroscopic. Next, we bring the system to air ambient pressure and tempearture (~ 20 °C) and measure the ionic gate current as a function of the ionic gate voltage was measured for various scan rates, ν. These curves are plotted as CV curves in

Supplementary Figure 8b. The capacitance was extracted from area enclosed by the CV curves ($\int IdV$) via:

$$C_{Meas} = \frac{1}{2\Delta V \nu} \int IdV$$

Where ΔV=0.2 V is the scan range and the factor of 2 comes from the fact that only half the area enclosed by the CV curve is associated with charging (the other half is discharging).

We note that this measured capacitance includes a small parallel background capacitance associated with double layer formation on the electrodes. This is given by

$$C_{BG} = \varepsilon_r \varepsilon_0 A_{electrodes} / \lambda$$

where λ (usually taken as 1 nm) is the double layer thickness and ε_r is usually taken as 10.[15] Here the electrode area is $A_{electrodes}$=0.75 mm^2.

The channel capacitance (C) is given by $C = C_{Meas} - C_{BG}$. This value was then normalised to the channel area (0.0055 cm^2, calculated from the product of channel length, 50 μm and width 11 mm) and plotted versus scan rate in Supplementary Figure 8b. This data shows the areal capacitance to fall off with increasing scan rate as is commonly seen for supercapacitors.[16]

We can check this data by noting that this dependence of capacitance on scan rate is described by[16]

$$C = C_{Low-rate} \left[1 - \frac{\nu\tau}{\Delta V} \left(1 - e^{-\Delta V/\nu\tau} \right) \right]$$

Where τ is the characteristic time associated with charge or discharge. The solid line in Supplementary Figure 8b is a fit to this data which shows very good agreement with the data. The fit gives a value of the capacitance at infinitely low rate of $C_{Low-rate}$=28 μF/cm^2. This value is a measure fo the capacitance unhindered by rate effects and so can be compared to theory.

When $C_{Low\text{-}rate}$ is normalised to area, it is equal to the product of the low-rate volumetric capacitance of the device (C_V) times the device thickness (25 nm). For a nanosheet network, it has been shown that[4]

$$C_V = \frac{2\varepsilon_r\varepsilon_0(1-P)}{\lambda\langle t\rangle}$$

where P is the network porosity and $\langle t \rangle$ is the mean nanosheet thickness (λ and ε_r have the same values as above). Taking $\langle t \rangle$=10 nm (see AFM data) and assuming a porosity of 30%, we find C_V=12.4 F/cm^3. Taking a device thickness of 25 nm yields a predicted low-rate areal capacitance of 3.1 μF/cm^2. This is very close to the value of $C_{Low\text{-}rate}$=2.8 μF/cm^2 extracted from fitting the data above which shows that the capacitances values in Supplementary Figure 8b are in line with expectations. Then, taking v at 50 mV/s (in line with scan rates used in our transfer and output characteristics) we estimate the C_{device} as ~ 3.1 μF cm^{-2} comparable to previous reports.[17-19] The value of C_{device} need to be scaled for our WS_2, WSe_2 and MoS_2 devices used in Figure 2 and 5 for the main text respectively to account for the different network thicknesses shown in Supplementary Figure 5. We find C_{device} of ~ 3.1 μF cm^{-2} and ~ 4.9 μF cm^{-2} for our WS_2 and WSe_2 devices respectively. We also find C_{device} of ~ 3.1 μF cm^{-2}, ~ 3.1 μF cm^{-2} and ~ 1.2 μF cm^{-2} for our MoS_2 networks of $\langle L \rangle$ ~ 1040, $\langle L \rangle$ ~ 605 nm and $\langle L \rangle$ ~ 484 nm used in our $\langle L \rangle$ dependence study in Figure 4 (main text).

Supplementary Note 7

Literature review of TMD transistors with solution processed networks

Summary of TMD network transistor literature							
			Acid Treatment or Vacuum Measurement	No Treatment			
Material	Substrate	Annealing Temp (°C)	μ (cm^2 V^{-1} s^{-1})	μ (cm^2 V^{-1} s^{-1})	I_{on}/I_{off}	SS (mV/dec)	Ref.
MoS_2	Si/SiO_2	300	7		10^6	~6000	20
MoS_2	Si/SiO_2	450		0.00048	3	>3000	21
MoS_2	Si/SiO_2	350	0.4		10^6	~5000	22

MoS_2	PET	70	0.15		100	>500	17
WSe_2	PET	70	0.08		100	>500	17
WS_2	PET	70	0.22		100	>500	17
$MoSe_2$	PET	70	0.18		100	>500	17
MoS_2	PET	50		0.3	2	>10000	23
MoS_2	Glass	250		0.02			24
MoS_2	Si/SiO_2	400		0.1	25	~800	18
MoS_2	Si/SiO_2	400	0.06		50	770	25
WS_2	Glass	100	0.01		10^4	~250	26
MoS_2	Si/SiO_2	110	0.01		10^4	~500	27
MoS_2	Si/SiO_2	200		0.73	10^5	~5000	28
MoS_2	Si/SiO_2	200		0.0004	22	>40000	29
WSe_2	PET	80	0.08		10^3	~700	19
WS_2	PET	80	0.1		10^3	~700	19
MoS_2	Si/SiO_2	80	1.8		10^6	~3000	30
MoS_2	Si/SiO_2	80	8.1		10^2	~20000	30
MoS_2	Si/SiO_2	1000		0.09	10^3	~10500	31
MoS_2	Si/SiO_2	1000		0.008	10^2	~17300	31
MoS_2	Si/SiO_2	1000		7.9	10^5	~2300	31
MoS_2	Si/SiO_2	1000		2.1	10^4	~6600	31
MoS_2	Si/SiO_2	300	8.3		10^6	~250	32
MoS_2	Si/SiO_2	200	0.2		14	~40,000	33
MoS_2	Si/SiO_2	400	5		10^6	~1000	34
MoS_2	(Leaf/Skin) Si/SiO_2	300	10		100	~2000	35
MoS_2	Si/SiO_2	500	3.8		10^5	200	36
Our MoS_2	Si/SiO_2	120		11	10^3	542	Ours
Our WSe_2	Si/SiO_2	120		2	10^3	182	Ours
Our WS_2	Si/SiO_2	120		9	10^4	339	Ours
Our WSe_2	PET	120		2.8	10^3	300	Ours

Supplementary Table 1: Table summarising literature on solution processed TMD networks.

Type	Semiconductor	μ_p (cm^2 V^{-1} s^{-1})	μ_n (cm^2 V^{-1} s^{-1})	I_{on}/I_{off}	Ref.
**Organic**	PhC2−BQQDI		3	10^7	37
**Organic**	C6–DNT–VW	1.2 – 9.5		10^5	38
**Organic**	Lisicon SP400	0.4		10^5	39
**Organic**	PC12TV12T (p), P(ND12OD-T2) (n)	0.5		3×10^6	40
**Organic**	PDVT-8:PS3	0.58		5×10^4	41
**Organic**	PDPPTzBT	1.8		10^8	42
**Organic**	poly(3-hexythiophene)	1.73		9×10^5	43
**Metal Oxide**	ZnO	1.48		4×10^5	43
**Metal Oxide**	a-InZnO		12.9	5×10^6	44
**Metal Oxide**	SnOx (p) GIZO (n)	1.2	23	10^4 (n) 10^2 (p)	45
**Metal Oxide**	IGZO		2	10^5	46
**Metal Oxide**	In_2O_3/IGZO		14.5	6.5×10^6	47
**Metal Oxide**	IGZO		0.52	4×10^4	48
**Metal Oxide**	InGaZnO		6-10	10^3	49
**Metal Oxide**	InSnO		3.14	5×10^8	50
**CNTs**	CNT	9	3	3×10^5	51
**CNTs**	CNT	31	17	3×10^3	51
**CNTs**	CNT	9		10^5	52

**CNTs**	CNT	3.92		10^4	53
**CNTs**	CNT	12.25		10^5	54
**CNTs**	CNT	3.6		10^3	55
**Graphene**	Graphene (semi-metal)	204	118	2.5	56
**Graphene**	Graphene (semi-metal)		95	10	57

Supplementary Table 2: Table summarising solution processing literature on organic polymers, metal oxides, graphene and CNTs used in figure 3c of the main text.

Supplementary References

1. Plechinger, G.; Heydrich, S.; Eroms, J.; Weiss, D.; Schüller, C.; Korn, T., Raman spectroscopy of the interlayer shear mode in few-layer MoS2 flakes. *Applied Physics Letters* **2012,** *101* (10), 101906.
2. Fujisawa, K.; Carvalho, B. R.; Zhang, T.; Perea-Lopez, N.; Lin, Z.; Carozo, V.; Ramos, S.; Kahn, E.; Bolotsky, A.; Liu, H.; Elias, A. L.; Terrones, M., Quantification and Healing of Defects in Atomically Thin Molybdenum Disulfide: Beyond the Controlled Creation of Atomic Defects. *ACS Nano* **2021,** *15* (6), 9658-9669.
3. Wang, F.; Kinloch, I. A.; Wolverson, D.; Tenne, R.; Zak, A.; O'Connell, E.; Bangert, U.; Young, R. J., Strain-induced phonon shifts in tungsten disulfide nanoplatelets and nanotubes. *2D Mater.* **2016,** *4* (1), 015007.
4. Zhang, R.; Drysdale, D.; Koutsos, V.; Cheung, R., Controlled Layer Thinning and p-Type Doping of WSe2 by Vapor XeF2. *Adv. Funct. Mater.* **2017,** *27* (41), 1702455.
5. Zhao, W.; Ghorannevis, Z.; Amara, K. K.; Pang, J. R.; Toh, M.; Zhang, X.; Kloc, C.; Tan, P. H.; Eda, G., Lattice dynamics in mono- and few-layer sheets of WS2 and WSe2. *Nanoscale* **2013,** *5* (20), 9677-83.
6. Er, E.; Hou, H.-L.; Criado, A.; Langer, J.; Möller, M.; Erk, N.; Liz-Marzán, L. M.; Prato, M., High-Yield Preparation of Exfoliated 1T-MoS2 with SERS Activity. *Chemistry of Materials* **2019,** *31* (15), 5725-5734.
7. Han, A.; Zhou, X.; Wang, X.; Liu, S.; Xiong, Q.; Zhang, Q.; Gu, L.; Zhuang, Z.; Zhang, W.; Li, F.; Wang, D.; Li, L.-J.; Li, Y., One-step synthesis of single-site vanadium substitution in 1T-WS2 monolayers for enhanced hydrogen evolution catalysis. *Nat. Commun* **2021,** *12* (1), 709.
8. Calandra, M., Chemically exfoliated single-layer MoS_2: Stability, lattice dynamics, and catalytic adsorption from first principles. *Phys. Rev. B* **2013,** *88* (24), 245428.
9. Sokolikova, M. S.; Sherrell, P. C.; Palczynski, P.; Bemmer, V. L.; Mattevi, C., Direct solution-phase synthesis of 1T' WSe2 nanosheets. *Nat. Commun* **2019,** *10* (1), 712.
10. Kelly, A. G.; O'Suilleabhain, D.; Gabbett, C.; Coleman, J. N., The electrical conductivity of solution-processed nanosheet networks. *Nature Reviews Materials* **2021,** *7* (3), 217-234.
11. Backes, C.; Smith, R. J.; McEvoy, N.; Berner, N. C.; McCloskey, D.; Nerl, H. C.; O'Neill, A.; King, P. J.; Higgins, T.; Hanlon, D.; Scheuschner, N.; Maultzsch, J.; Houben, L.; Duesberg, G. S.; Donegan, J. F.; Nicolosi, V.; Coleman, J. N., Edge and confinement effects allow in situ measurement of size and thickness of liquid-exfoliated nanosheets. *Nat Commun* **2014,** *5* (1), 4576.
12. Backes, C.; Szydlowska, B. M.; Harvey, A.; Yuan, S.; Vega-Mayoral, V.; Davies, B. R.; Zhao, P. L.; Hanlon, D.; Santos, E. J.; Katsnelson, M. I.; Blau, W. J.; Gadermaier, C.; Coleman, J. N., Production of Highly Monolayer Enriched Dispersions of Liquid-Exfoliated Nanosheets by Liquid Cascade Centrifugation. *ACS Nano* **2016,** *10* (1), 1589-601.
13. Kaphle, V.; Liu, S.; Keum, C.-M.; Lüssem, B., Organic Electrochemical Transistors Based on Room Temperature Ionic Liquids: Performance and Stability. *physica status solidi (a)* **2018,** *215* (24), 1800631.

14. Bu, X.; Xu, H.; Shang, D.; Li, Y.; Lv, H.; Liu, Q., Ion-Gated Transistor: An Enabler for Sensing and Computing Integration. *Advanced Intelligent Systems* **2020,** *2* (12), 2000156.
15. Gholamvand, Z.; McAteer, D.; Harvey, A.; Backes, C.; Coleman, J. N., Electrochemical Applications of Two-Dimensional Nanosheets: The Effect of Nanosheet Length and Thickness. *Chemistry of Materials* **2016,** *28* (8), 2641-2651.
16. Higgins, T. M.; Coleman, J. N., Avoiding Resistance Limitations in High-Performance Transparent Supercapacitor Electrodes Based on Large-Area, High-Conductivity PEDOT:PSS Films. *ACS Applied Materials & Interfaces* **2015,** *7* (30), 16495-16506.
17. Kelly, A. G.; Hallam, T.; Backes, C.; Harvey, A.; Esmaeily, A. S.; Godwin, I.; Coelho, J.; Nicolosi, V.; Lauth, J.; Kulkarni, A.; Kinge, S.; Siebbeles, L. D.; Duesberg, G. S.; Coleman, J. N., All-printed thin-film transistors from networks of liquid-exfoliated nanosheets. *Science* **2017,** *356* (6333), 69-73.
18. Piatti, E.; Arbab, A.; Galanti, F.; Carey, T.; Anzi, L.; Spurling, D.; Roy, A.; Zhussupbekova, A.; Patel, K. A.; Kim, J. M.; Daghero, D.; Sordan, R.; Nicolosi, V.; Gonnelli, R. S.; Torrisi, F., Charge transport mechanisms in inkjet-printed thin-film transistors based on two-dimensional materials. *Nature Electronics* **2021,** *4* (12), 893-905.
19. O'Suilleabhain, D.; Kelly, A. G.; Tian, R.; Gabbett, C.; Horvath, D.; Coleman, J. N., Effect of the Gate Volume on the Performance of Printed Nanosheet Network-Based Transistors. *ACS Applied Electronic Materials* **2020,** *2* (7), 2164-2170.
20. Lin, Z.; Liu, Y.; Halim, U.; Ding, M.; Liu, Y.; Wang, Y.; Jia, C.; Chen, P.; Duan, X.; Wang, C.; Song, F.; Li, M.; Wan, C.; Huang, Y.; Duan, X., Solution-processable 2D semiconductors for high-performance large-area electronics. *Nature* **2018,** *562* (7726), 254-258.
21. Li, J.; Naiini, M. M.; Vaziri, S.; Lemme, M. C.; Östling, M., Inkjet Printing of MoS2. *Adv. Funct. Mater.* **2014,** *24* (41), 6524-6531.
22. Xi, Y.; Serna, M. I.; Cheng, L.; Gao, Y.; Baniasadi, M.; Rodriguez-Davila, R.; Kim, J.; Quevedo-Lopez, M. A.; Minary-Jolandan, M., Fabrication of MoS2 thin film transistors via selective-area solution deposition methods. *J. Mater. Chem. C* **2015,** *3* (16), 3842-3847.
23. He, Q.; Zeng, Z.; Yin, Z.; Li, H.; Wu, S.; Huang, X.; Zhang, H., Fabrication of flexible MoS2 thin-film transistor arrays for practical gas-sensing applications. *Small* **2012,** *8* (19), 2994-9.
24. Yu, X.; Prévot, M. S.; Sivula, K., Multiflake Thin Film Electronic Devices of Solution Processed 2D MoS2 Enabled by Sonopolymer Assisted Exfoliation and Surface Modification. *Chemistry of Materials* **2014,** *26* (20), 5892-5899.
25. Carey, T.; Arbab, A.; Anzi, L.; Bristow, H.; Hui, F.; Bohm, S.; Wyatt-Moon, G.; Flewitt, A.; Wadsworth, A.; Gasparini, N.; Kim, J. M.; Lanza, M.; McCulloch, I.; Sordan, R.; Torrisi, F., Inkjet Printed Circuits with 2D Semiconductor Inks for High-Performance Electronics. *Adv. Electron. Mater.* **2021,** *7* (7), 2100112.
26. Higgins, T. M.; Finn, S.; Matthiesen, M.; Grieger, S.; Synnatschke, K.; Brohmann, M.; Rother, M.; Backes, C.; Zaumseil, J., Electrolyte-Gated n-Type Transistors Produced from Aqueous Inks of WS2 Nanosheets. *Adv. Funct. Mater.* **2019,** *29* (4), 1804387.
27. Ippolito, S.; Kelly, A. G.; Furlan de Oliveira, R.; Stoeckel, M. A.; Iglesias, D.; Roy, A.; Downing, C.; Bian, Z.; Lombardi, L.; Samad, Y. A.; Nicolosi, V.; Ferrari, A. C.; Coleman, J. N.; Samori, P., Covalently interconnected transition metal dichalcogenide networks via defect engineering for high-performance electronic devices. *Nat Nanotechnol* **2021,** *16* (5), 592-598.
28. Neilson, J.; Avery, M. P.; Derby, B., Tiled Monolayer Films of 2D Molybdenum Disulfide Nanoflakes Assembled at Liquid/Liquid Interfaces. *ACS Appl. Mater. Interfaces* **2020,** *12* (22), 25125-25134.
29. Zeng, X.; Hirwa, H.; Metel, S.; Nicolosi, V.; Wagner, V., Solution processed thin film transistor from liquid phase exfoliated MoS2 flakes. *Solid-State Electronics* **2018,** *141*, 58-64.
30. Kim, J.; Jung, M.; Lim, D. U.; Rhee, D.; Jung, S. H.; Cho, H. K.; Kim, H. K.; Cho, J. H.; Kang, J., Area-Selective Chemical Doping on Solution-Processed MoS2 Thin-Film for Multi-Valued Logic Gates. *Nano Lett* **2022,** *22* (2), 570-577.

31. Kwack, Y.-J.; Can, T. T. T.; Choi, W.-S., Bottom-up water-based solution synthesis for a large MoS2 atomic layer for thin-film transistor applications. *npj 2D Materials and Applications* **2021,** *5* (1), 84.
32. Kim, J.; Rhee, D.; Song, O.; Kim, M.; Kwon, Y. H.; Lim, D. U.; Kim, I. S.; Mazanek, V.; Valdman, L.; Sofer, Z.; Cho, J. H.; Kang, J., All-Solution-Processed Van der Waals Heterostructures for Wafer-Scale Electronics. *Adv Mater* **2022,** *34* (12), e2106110.
33. Wells, R. A.; Zhang, M.; Chen, T. H.; Boureau, V.; Caretti, M.; Liu, Y.; Yum, J. H.; Johnson, H.; Kinge, S.; Radenovic, A.; Sivula, K., High Performance Semiconducting Nanosheets via a Scalable Powder-Based Electrochemical Exfoliation Technique. *ACS Nano* **2022,** *16* (4), 5719-5730.
34. Ma, C.; Xu, D.; Wang, P.; Lin, Z.; Zhou, J.; Jia, C.; Huang, J.; Li, S.; Huang, Y.; Duan, X., Two-dimensional van der Waals thin film transistors as active matrix for spatially resolved pressure sensing. *Nano Res.* **2021,** *14* (10), 3395-3401.
35. Yan, Z.; Xu, D.; Lin, Z.; Wang, P.; Cao, B.; Ren, H.; Song, F.; Wan, C.; Wang, L.; Zhou, J.; Zhao, X.; Chen, J.; Huang, Y.; Duan, X., Highly stretchable van der Waals thin films for adaptable and breathable electronic membranes. *Science* **2022,** *375* (6583), 852-859.
36. Song, O.; Rhee, D.; Kim, J.; Jeon, Y.; Mazánek, V.; Söll, A.; Kwon, Y. A.; Cho, J. H.; Kim, Y.-H.; Sofer, Z.; Kang, J., All inkjet-printed electronics based on electrochemically exfoliated two-dimensional metal, semiconductor, and dielectric. *npj 2D Materials and Applications* **2022,** *6* (1), 64.
37. Okamoto, T.; Kumagai, S.; Fukuzaki, E.; Ishii, H.; Watanabe, G.; Niitsu, N.; Annaka, T.; Yamagishi, M.; Tani, Y.; Sugiura, H.; Watanabe, T.; Watanabe, S.; Takeya, J., Robust, high-performance n-type organic semiconductors. *Sci Adv* **2020,** *6* (18), eaaz0632.
38. Okamoto, T.; Mitsui, C.; Yamagishi, M.; Nakahara, K.; Soeda, J.; Hirose, Y.; Miwa, K.; Sato, H.; Yamano, A.; Matsushita, T.; Uemura, T.; Takeya, J., V-shaped organic semiconductors with solution processability, high mobility, and high thermal durability. *Adv Mater* **2013,** *25* (44), 6392-7.
39. Grau, G.; Subramanian, V., Fully High-Speed Gravure Printed, Low-Variability, High-Performance Organic Polymer Transistors with Sub-5 V Operation. *Adv. Electron. Mater.* **2016,** *2* (4), 1500328.
40. Baeg, K.-J.; Khim, D.; Kim, J.; Kim, D.-Y.; Sung, S.-W.; Yang, B.-D.; Noh, Y.-Y., Flexible Complementary Logic Gates Using Inkjet-Printed Polymer Field-Effect Transistors. *IEEE Electron Device Letters* **2013,** *34* (1), 126-128.
41. Yang, H.; Zhang, G.; Zhu, J.; He, W.; Lan, S.; Liao, L.; Chen, H.; Guo, T., Improving Charge Mobility of Polymer Transistors by Judicious Choice of the Molecular Weight of Insulating Polymer Additive. *The Journal of Physical Chemistry C* **2016,** *120* (31), 17282-17289.
42. Wang, H.; Cheng, C.; Zhang, L.; Liu, H.; Zhao, Y.; Guo, Y.; Hu, W.; Yu, G.; Liu, Y., Inkjet printing short-channel polymer transistors with high-performance and ultrahigh photoresponsivity. *Adv Mater* **2014,** *26* (27), 4683-9.
43. Hong, K.; Kim, S. H.; Mahajan, A.; Frisbie, C. D., Aerosol jet printed p- and n-type electrolyte-gated transistors with a variety of electrode materials: exploring practical routes to printed electronics. *ACS Appl. Mater. Interfaces* **2014,** *6* (21), 18704-11.
44. Kaneda, T.; Hirose, D.; Miyasako, T.; Tue, P. T.; Murakami, Y.; Kohara, S.; Li, J.; Mitani, T.; Tokumitsu, E.; Shimoda, T., Rheology printing for metal-oxide patterns and devices. *J. Mater. Chem. C* **2014,** *2* (1), 40-49.
45. Martins, R.; Nathan, A.; Barros, R.; Pereira, L.; Barquinha, P.; Correia, N.; Costa, R.; Ahnood, A.; Ferreira, I.; Fortunato, E., Complementary metal oxide semiconductor technology with and on paper. *Adv Mater* **2011,** *23* (39), 4491-6.
46. Martins, R.; Gaspar, D.; Mendes, M. J.; Pereira, L.; Martins, J.; Bahubalindruni, P.; Barquinha, P.; Fortunato, E., Papertronics: Multigate paper transistor for multifunction applications. *Applied Materials Today* **2018,** *12*, 402-414.
47. Liang, K.; Wang, Y.; Shao, S.; Luo, M.; Pecunia, V.; Shao, L.; Zhao, J.; Chen, Z.; Mo, L.; Cui, Z., High-performance metal-oxide thin-film transistors based on inkjet-printed self-confined bilayer heterojunction channels. *J. Mater. Chem. C* **2019,** *7* (20), 6169-6177.

48. Wang, B.; Thukral, A.; Xie, Z.; Liu, L.; Zhang, X.; Huang, W.; Yu, X.; Yu, C.; Marks, T. J.; Facchetti, A., Flexible and stretchable metal oxide nanofiber networks for multimodal and monolithically integrated wearable electronics. *Nat Commun* **2020,** *11* (1), 2405.
49. Nomura, K.; Ohta, H.; Takagi, A.; Kamiya, T.; Hirano, M.; Hosono, H., Room-temperature fabrication of transparent flexible thin-film transistors using amorphous oxide semiconductors. *Nature* **2004,** *432* (7016), 488-92.
50. Hwan Hwang, Y.; Seo, J.-S.; Moon Yun, J.; Park, H.; Yang, S.; Ko Park, S.-H.; Bae, B.-S., An 'aqueous route' for the fabrication of low-temperature-processable oxide flexible transparent thin-film transistors on plastic substrates. *NPG Asia Materials* **2013,** *5* (4), e45-e45.
51. Ha, M.; Xia, Y.; Green, A. A.; Zhang, W.; Renn, M. J.; Kim, C. H.; Hersam, M. C.; Frisbie, C. D., Printed, sub-3V digital circuits on plastic from aqueous carbon nanotube inks. *ACS Nano* **2010,** *4* (8), 4388-95.
52. Lau, P. H.; Takei, K.; Wang, C.; Ju, Y.; Kim, J.; Yu, Z.; Takahashi, T.; Cho, G.; Javey, A., Fully printed, high performance carbon nanotube thin-film transistors on flexible substrates. *Nano Lett* **2013,** *13* (8), 3864-9.
53. Cao, X.; Lau, C.; Liu, Y.; Wu, F.; Gui, H.; Liu, Q.; Ma, Y.; Wan, H.; Amer, M. R.; Zhou, C., Fully Screen-Printed, Large-Area, and Flexible Active-Matrix Electrochromic Displays Using Carbon Nanotube Thin-Film Transistors. *ACS Nano* **2016,** *10* (11), 9816-9822.
54. Cardenas, J. A.; Catenacci, M. J.; Andrews, J. B.; Williams, N. X.; Wiley, B. J.; Franklin, A. D., In-Place Printing of Carbon Nanotube Transistors at Low Temperature. *ACS Applied Nano Materials* **2018,** *1* (4), 1863-1869.
55. Numata, H.; Ihara, K.; Saito, T.; Endoh, H.; Nihey, F., Highly Uniform Thin-Film Transistors Printed on Flexible Plastic Films with Morphology-Controlled Carbon Nanotube Network Channels. *Applied Physics Express* **2012,** *5* (5), 055102.
56. Carey, T.; Cacovich, S.; Divitini, G.; Ren, J.; Mansouri, A.; Kim, J. M.; Wang, C.; Ducati, C.; Sordan, R.; Torrisi, F., Fully inkjet-printed two-dimensional material field-effect heterojunctions for wearable and textile electronics. *Nat Commun* **2017,** *8* (1), 1202.
57. Torrisi, F.; Hasan, T.; Wu, W.; Sun, Z.; Lombardo, A.; Kulmala, T. S.; Hsieh, G. W.; Jung, S.; Bonaccorso, F.; Paul, P. J.; Chu, D.; Ferrari, A. C., Inkjet-printed graphene electronics. *ACS Nano* **2012,** *6* (4), 2992-3006.
